# Adjustment of photosynthetic activity to drought and fluctuating light in wheat

**DOI:** 10.1111/pce.13756

**Published:** 2020-03-27

**Authors:** Michele Grieco, Valentin Roustan, Georgi Dermendjiev, Sanna Rantala, Arpit Jain, Manuela Leonardelli, Kerstin Neumann, Vitus Berger, Doris Engelmeier, Gert Bachmann, Ingo Ebersberger, Eva‐Mari Aro, Wolfram Weckwerth, Markus Teige

**Affiliations:** ^1^ Ecogenomics and Systems Biology University of Vienna Vienna Austria; ^2^ Department of Genebank, Leibniz Institute of Plant Genetics and Crop Plant Research (IPK) Seeland Germany; ^3^ Molecular Plant Biology University of Turku Turku Finland; ^4^ Applied Bioinformatics Group Institute of Cell Biology and Neuroscience, Goethe‐University Frankfurt Frankfurt Germany; ^5^ Senckenberg Biodiversity and Climate Research Centre (S‐BIK‐F) Frankfurt Germany; ^6^ LOEWE Center for Translational Biodiversity Genomics Frankfurt Germany; ^7^ Vienna Metabolomics Center (VIME) University of Vienna Vienna Austria; ^8^ Max Perutz Labs, Department of Biochemistry & Cell Biology University of Vienna Vienna Austria

**Keywords:** breeding, crop, drought, light harvesting complex II, NPQ, photosynthesis, photosystem II, protein phosphorylation, wheat

## Abstract

Drought is a major cause of losses in crop yield. Under field conditions, plants exposed to drought are usually also experiencing rapid changes in light intensity. Accordingly, plants need to acclimate to both, drought and light stress. Two crucial mechanisms in plant acclimation to changes in light conditions comprise thylakoid protein phosphorylation and dissipation of light energy as heat by non‐photochemical quenching (NPQ). Here, we analyzed the acclimation efficacy of two different wheat varieties, by applying fluctuating light for analysis of plants, which had been subjected to a slowly developing drought stress as it usually occurs in the field. This novel approach allowed us to distinguish four drought phases, which are critical for grain yield, and to discover acclimatory responses which are independent of photodamage. In short‐term, under fluctuating light, the slowdown of NPQ relaxation adjusts the photosynthetic activity to the reduced metabolic capacity. In long‐term, the photosynthetic machinery acquires a drought‐specific configuration by changing the PSII‐LHCII phosphorylation pattern together with protein stoichiometry. Therefore, the fine‐tuning of NPQ relaxation and PSII‐LHCII phosphorylation pattern represent promising traits for future crop breeding strategies.

## Abstract 2

Elucidating the regulation of photosynthesis under the combination of abiotic stress and fluctuating light is crucial for understanding the plant physiology in realistic conditions and for crop improvement. Wheat copes with drought by reconfiguring the photosynthetic machinery and by slowing down the NPQ relaxation in fluctuating light before photodamage becomes detectable.

## 1 INTRODUCTION

Drought stress is one of the major limitations of plant growth and causes globally severe losses of crop yield, particularly for cereals. Drought already caused average yield reductions of 9–10% across the globe between 1964 and 2007, and current climate models predict further worsening of this situation (Lesk, Rowhani, & Ramankutty, 2016; Liu et al., 2016). Consequently, drought stress tolerance has been recognized as a prime target for future breeding programs (Araus, Slafer, Reynolds, & Royo, 2002; Ghatak, Chaturvedi, & Weckwerth, 2017; Hu & Xiong, 2014; Mittler & Blumwald, 2010). Drought stress poses a dilemma for plants: on the one hand, they need to close their stomata to avoid too much water loss, while on the other hand, they need to keep their stomata open for cooling and CO_2_ uptake (Chaves et al., 2016). Accordingly, photosynthetic activity becomes highly affected when plants have to react to drought. Drought constraints to photosynthesis have been thoroughly reviewed (Basu, Ramegowda, Kumar, & Pereira, 2016; Chaves, Flexas, & Pinheiro, 2009; Lawlor & Tezara, 2009; Pinheiro & Chaves, 2011). However, the relative importance of the different factors limiting photosynthesis is still controversial. Clearly, limiting CO_2_ availability due to decreased stomatal conductance is generally accepted as the main cause for decreased photosynthesis under mild to moderate water limitation (Pinheiro & Chaves, 2011). Importantly, under field conditions stomata will be closed particularly at midday when the light intensity becomes maximal, resulting in an excess of incident energy relative to the available intercellular CO_2_. Therefore, the amount of harvested light energy and generated reducing power can easily exceed the rate of its consumption by the Calvin‐Benson‐Bassham (CBB) cycle, thus causing excess of reactive oxygen species (ROS) and consequent cellular damage. Hence, plants employ a number of different protection mechanisms to mediate the dissipation of excess energy from light‐harvesting under CO_2_‐limiting conditions (Lawlor & Tezara, 2009). One strategy is a regulated thermal dissipation of excess energy within the light‐harvesting complexes involving the carotenoids of the xanthophyll cycle (Jahns & Holzwarth, 2012). Such photoprotective mechanisms compete with photochemistry for the absorbed energy and have therefore been denoted as non‐photochemical quenching (NPQ) of excitation energy (Bilger & Björkman, 1990). This leads to downregulation of photosynthesis, which can be measured as decrease in quantum yield of photosystem II (PSII) in chlorophyll fluorescence measurements (Murchie & Lawson, 2013).

In field conditions, plants that are subjected to drought are unavoidably forced to cope also with fast fluctuations of light intensity, due to clouds or leaf shading. It was shown that NPQ dynamics under fluctuating light (FL) is crucial for crop productivity. The rate of NPQ relaxation can account for up to 15% of crop yield (Kromdijk et al., 2016). Transgenic tobacco plants that were able to accelerate NPQ relaxation upon shift from high‐ to low‐light intensity showed increased biomass accumulation under field conditions. However, this achievement was obtained in the absence of a particular stress. This led to the important question of whether NPQ relaxation could constitute a critical regulatory point for crop productivity under stress conditions (Kaiser, Morales, & Harbinson, 2018; Slattery, Walker, Weber, & Ort, 2018). The term NPQ refers to different molecular mechanisms, which have not yet been fully elucidated. NPQ kinetics includes at least three components: energy‐dependent quenching (qE) is the fastest component activating and relaxing in the time scale of seconds; zeaxanthin‐dependent quenching (qZ) in the order of minutes; and inhibition‐quenching (qI) showing much slower kinetics (Nilkens et al., 2010). It should be noted that the labeling of NPQ components is a simplification, as the molecular mechanisms determining NPQ kinetics may interact with each other and with common factors. Zeaxanthin, for instance, could be involved in more than one component (Kress & Jahns, 2017). How these components change under drought has not been well investigated.

Studying the acclimation processes employed by plants under combined drought‐ and FL stress is challenging; however, it is necessary for improving our understanding of physiology under realistic environmental conditions. One of the difficulties in studying combined drought‐FL stress is the very different time scale at which effects of these two stresses appear. Under drought in field conditions, soil dries out in the time scale of weeks. Consequently, plants have time for adopting morphological and molecular changes. Under FL, on the contrary, the plant needs to react in the time scale of seconds or minutes. Under drought, FL constitutes even a higher stress compared to well‐watered plants because the limited intracellular CO_2_ availability due to closed stomata does further limit the plant's ability to cope with higher light intensity.

The interaction between drought and FL effects is therefore very complex. In order to dissect the effects of drought and FL, we applied the following experimental setup to wheat (*Triticum aestivum*). First, plants were subjected to a slowly‐developing drought (similar to what may occur in field conditions) under natural light in greenhouse. Here the monitoring of water use and NPQ allowed distinguishing four drought phases. Subsequently, we applied a similar drought treatment in a climate chamber, where plants were grown under controlled light conditions until a phase of drought was achieved in which they did not yet show symptoms of long‐term molecular damage. At different time points during the drought treatment, we applied a method, denoted here as “FL analysis” (fluctuating light analysis), on single leaves for a few minutes, while simultaneously monitoring the photosynthetic activity. This method was used to challenge the photosynthetic regulatory mechanisms under a fluctuation of light intensity and at the same time to quantify consequent photodamage. In other words, the growth light level served as a baseline, on which we applied FL for a few minutes and evaluated the FL‐induced photodamage during drought progression. As our aim was to understand the basic acclimation processes, we chose two spring wheat cultivars originated from very different environments, with the purpose of identifying common response mechanisms implemented by both cultivars. FL analysis in the chamber experiments revealed the same drought phases as observed in the greenhouse experiment before. Moreover, the “FL analysis” uncovered also changes in the NPQ kinetics. This implies that stressed wheat plants employed drought‐dependent long‐term modifications that had effects also in the time scale of fast light changes. Drought‐stressed plants actually exhibited a reconfiguration of the photosynthetic machinery, consisting in the modification of the photosynthetic enzymes stoichiometry and in the acquisition of a long‐term PSII and Light Harvesting Complex II (LHCII) phosphorylation pattern. These structural and functional changes constitute active regulatory mechanisms because they were independent from both short‐term and long‐term drought‐induced damage. Finally, we integrated these results to develop a model of the photosynthetic acclimation of wheat under water deficit. Suggestions are put forward for developing new traits to be applied to crop breeding.

## 2 MATERIALS AND METHODS

### 2.1 Plant material and growth conditions

Seeds of two spring wheat cultivars were obtained from the IPK genebank, one originating from the United Kingdom, accession number TRI 5357 (here indicated as UK), the other from Iran, accession number TRI 5630 (indicated as IR). Seeds were sown directly to soil in cylindrical pots (30 cm height, 11 cm diameter). Soil mixture: 3 parts of potting compost (peat, humus), 2 parts of sand, 1 part of Styromull (Royal Brinkman, the Netherlands). After the seedling establishment, we thinned out to one plant per pot. Drought stress was applied to wheat plants at the age of 4 weeks, when they reached a developmental stage comprised in BBCH 23–33 phases (Witzenberger et al., 1989). In the chamber, plants were grown in controlled conditions: relative humidity was 55%; light was provided by metal halide lamps (HRI‐TS TS 250W/NDL Neutral White, Radium, Germany) at the intensity of 200 μmol photons m^−2^ s^−1^ from 7 a.m. to 9 a.m. and from 4 p.m. to 9 p.m., 450 μmol photons m^−2^ s^−1^ from 9 a.m. to 4 p.m. (14 hr day/10 hr night); temperature was 24°C in the day and 16°C at night. All measurements were carried out on leaves acclimated to the light phase of 450 μmol photons m^−2^ s^−1^. For in vitro analyses, all leaves were collected at 3 p.m. For in vivo analyses (applied on single leaves), leaves were chosen randomly.

The greenhouse experiment was carried out at IPK. Temperature remained in the range of 17–25°C during the day, and 17–19°C in the night; relative humidity in the range of 50–80%. Wheat plants were grown in 2 L pots. Watering and pot weighing were performed automatically by the phenotyping and imaging platform LemnaTec‐Scanalyzer 3D (LemnaTec GmbH, Aachen, Germany). When the internal sunlight intensity was lower than 200 μmol photons m^−2^ s^−1^, additional light was supplied by halogen lamps (at 200 μmol photons m^−2^ s^−1^) until a maximum of 15 h light per day.

### 2.2 Water deficit application and monitoring

In both greenhouse and chamber experiments, water deficit was applied by complete water withdrawal until the soil water content approached a plateau value, that is, at the exhaustion of extractable water. In greenhouse, soil water content was monitored by weighing the pots and quantified as percentage of soil saturation (% of field capacity). Subsequently, soil was rewatered and maintained at the well‐watered level (90% of field capacity) until harvest. In the chamber, soil dehydration was monitored by measuring the volumetric soil water content (volume of water/ total volume ratio, in percentage) through ML3 sensors (Delta‐T Devices, United Kingdom). Sensors were placed at the bottom of soil through a hole in the pot. When the soil water content reached the plateau value, leaves were collected and frozen in liquid nitrogen for further analysis. Leaf water content was estimated by the formula (FW − DW)/(TW − DW) × 100, where FW is the leaf fresh weigh, DW is the leaf dry weight, and TW is the leaf turgid weight. Leaves were cut at the basal portion, and the cut edge of leaf section was placed in distilled water (until about 2 mm depth) overnight at 4°C for allowing full leaf rehydration and the determination of TW. Subsequently, leaves were dried at 60°C for 4 days for the determination of DW. Stomata conductance was measured by PWMR‐4 porometer (PP Systems, USA) between 11 a.m. and 2 p.m.

### 2.3 Biochemical analysis

All leaves from every plant were collected. In order to exclude leaf extremities, only the central portion (approximately one‐third of leaf length) was collected and frozen in liquid nitrogen for biochemical analyses. Chlorophyll content per leaf area was determined on leaf discs (4 mm diameter) according to the method described in (Porra, Thompson, & Kriedemann, 1989).

### 2.4 Analysis of pigments and stress markers

Pigments were determined by HPLC analysis essentially according to (Czarnecki, Peter, & Grimm, 2011). Briefly, 2 mg of freeze‐dried leaf material was extracted with methanol/chloroform, and the extracts were separated on a Prontosil 200‐3‐C30 column (3 μm; 250 × 4.6 mm; Bischoff‐Chromatography) at 21°C with a flow rate of 0.2 ml min^−1^ using a Dionex Ultimate 3,000 UHPLC system. Elution was done with a gradient of solvent A (90% acetonitrile; 10% water; 0.1% triethylamine) and solvent B (100% ethyl acetate), and pigments were detected at 440 nm using a Dionex UVD 340U detector. Pigment standards for HPLC analysis were obtained from Extrasynthese S.A.S. FR (Genay, Cedex, FR; www.extrasynthese.com) for β‐carotene (Cat. No. 0303‐S), zeaxanthin (Cat. No. 0307‐S), and lutein (Cat. No. 0306‐S); and from DHI LAB Products (Horsholm, DK), for violaxanthin (Cat. No. PPS‐VIOL). The plant hormone abscisic acid (ABA) was detected using the ELISA kit from Cusabio (www.cusabio.com/ELISA-Kit/Plant-hormone-abscisic-acidABA-ELISA-Kit-62434.html). Briefly, 50 mg lyophilized leaf material where extracted with 700 μl sample extraction buffer (provided with kit) over night at 4°C, and the ABA content was measured photometrically at 450 nm using a standard dilution curve according to the manufacturer's instructions. Malondialdehyde (MDA) levels were quantified colorimetrically at 532 nm, using the thiobarbituric acid (TBA) method, which detects the MDA‐TB complex using a kit from abbexa (www.abbexa.com/lipid-peroxidation-mda-assay-kit). Therefore, 50 mg of lyophilized leaf material where extracted with 450 μl PBS buffer and colorimetrically quantified with thiobarbituric Acid (TBA) as MDA‐TB complex at 532 nm according to the manufacturer's protocol.

### 2.5 Thylakoid isolation

For the isolation of thylakoid membranes, leaves were mechanically ground in grinding buffer (50 mM HEPES–NaOH pH 7.5, 330 mM sorbitol, 5 mM MgCl_2_, 0.05% [wt/vol] BSA and 10 mM NaF), and the resulting suspension was filtered through Miracloth. Chloroplasts were collected by centrifugation (3,952 g for 7 min at 4°C), after which they were osmotically ruptured with shock buffer (50 mM HEPES–NaOH pH 7.5, 5 mM sorbitol, 10 mM MgCl_2_, and 10 mM NaF). The released thylakoid membranes were collected by centrifugation (3,952 g for 7 min at 4°C) and suspended in storage buffer (50 mM HEPES–NaOH pH 7.5, 100 mM sorbitol, 10 mM MgCl_2_, and 10 mM NaF). Chlorophyll concentration (Chl *a* + *b*) was determined according to Porra et al. (1989).

### 2.6 77 K fluorescence emission spectra

Fluorescence emission at the temperature of 77 K was recorded from isolated thylakoids with Ocean Optics S2000 spectrophotometer, using excitation wavelength of 480 nm. Thylakoid membranes were diluted to a concentration of 10 μg chl/ml with storage buffer (50 mM HEPES–NaOH pH 7.5, 100 mM sorbitol, 10 mM MgCl_2_, and 10 mM NaF).

### 2.7 Western blotting

Thylakoid membranes were dissolved to the concentration of 0.07 μg chl/μl with denaturating buffer (124 mM Tris–HCl pH 6.8, 5.4 M urea, 20% (vol/vol) glycerol, 4.3% (wt/vol) SDS, and 10% β‐mercaptoethanol) (Laemmli, 1970) and separated with SDS‐PAGE with 15% acrylamide and 6 M urea. For immunoblotting, proteins were transferred on PVDF membrane (Millipore). From the membrane, phosphorylated threonine residues were recognized with P‐Thr antibody (Cat. No. 6949S; New England Biolabs), while proteins D1, PSAB, LHCB2, and CP29 were detected with protein‐specific antibodies produced by Agrisera (Cat. No. AS10704, AS10695, AS01003, and AS04045, respectively). In detection, horseradish peroxidase‐linked secondary antibody (Agrisera) and Amersham ECL Western blotting detection reagents (GE Healthcare) were used. The membranes were subsequently stained with 0.1% Coomassie Brilliant Blue diluted in 40% methanol and 10% acetic acid.

### 2.8 Mass spectrometry

Total protein was extracted from wheat leaf extract and digested. Peptides were analyzed by LTQ‐Orbitrap Elite (Thermo). More information is available in Supporting information.

### 2.9 In vivo measurements of photosynthetic activity

**FIGURE 1:**
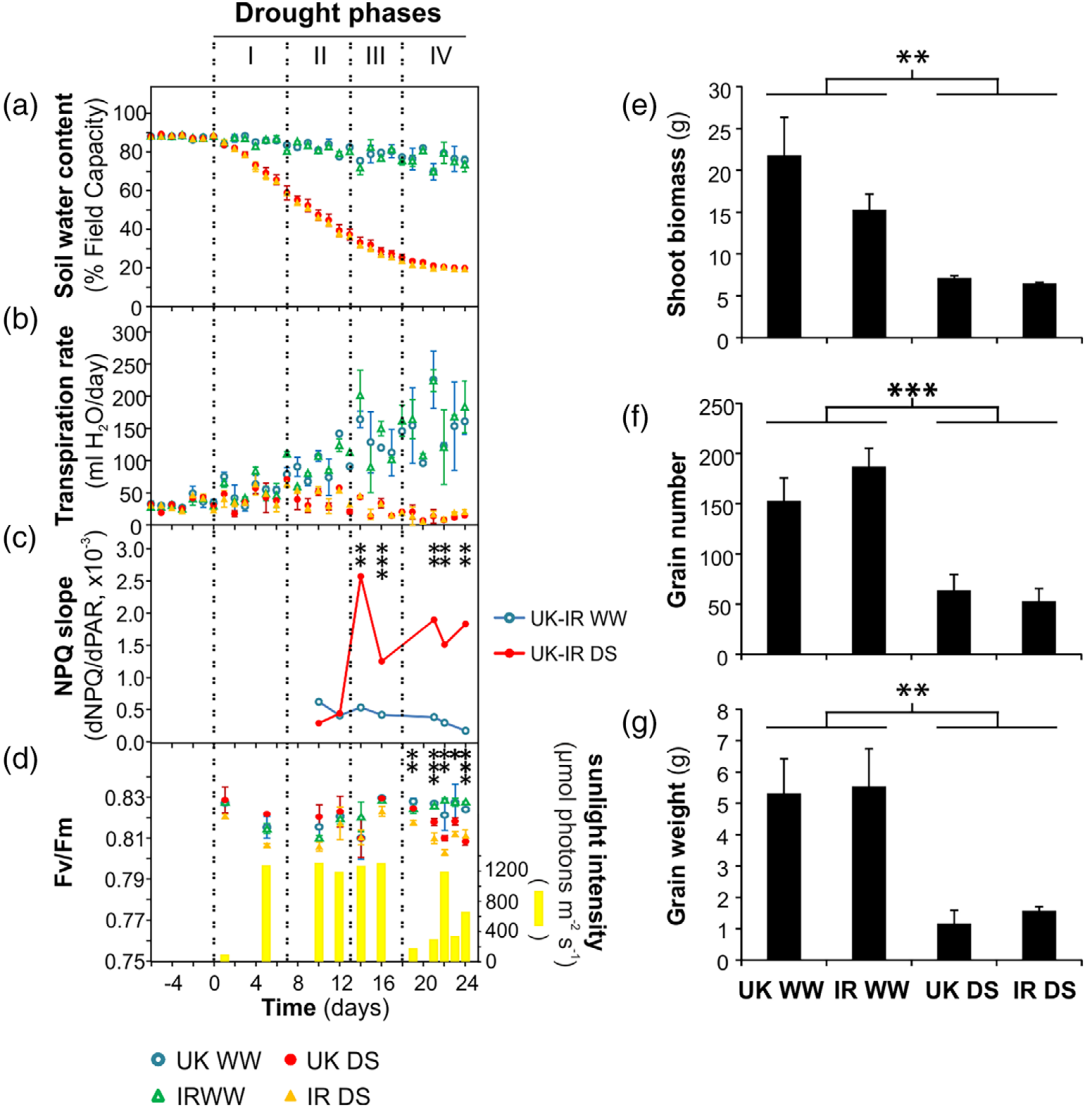
Drought phases in the United Kingdom (UK) and Iran (IR) wheat cultivars in well‐watered condition (WW) and in drought stress (DS) in the greenhouse experiment. “Time (days)” refers to the duration of drought treatment (day 0 denotes the last day of watering for treated plants). (a) Soil water content. (b) Transpiration rate. (c) Non‐photochemical quenching in relation to sunlight photosynthetically active radiation (PAR) reaching the leaf (see Figure S1 for more information). (d) Sunlight‐induced photodamage monitored by Fv/Fm parameter, and the outdoor sunlight intensity preceding the Fv/Fm measurement. (e‐g) Maturity data on the day of harvest. (e) Shoot biomass per plant. (f) Grain number per plant. (g) Total grain weight per plant. Data are means ± SD (*n* = 4). Asterisks indicate the p‐values of the control versus drought comparison (.01 < *p* < .05 is marked by *, .001 < *p* ≤ .01 by **, *p* < .001 by ***), estimated by Analysis of variance (except in c, estimated by ANCOVA)

**FIGURE 2:**
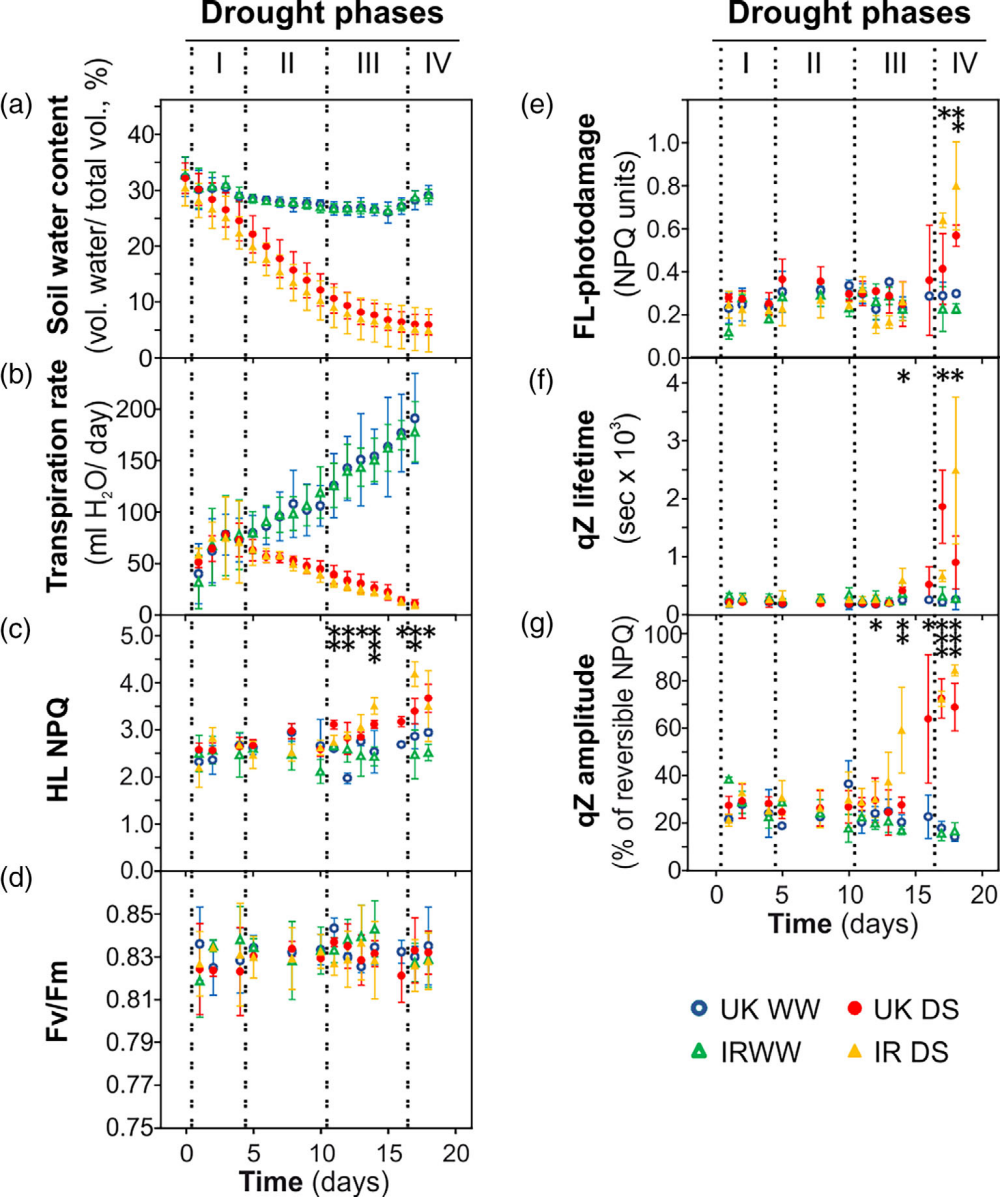
Drought phases in the United Kingdom (UK) and Iran (IR) wheat cultivars in well‐watered condition (WW) and in drought stress (DS) in chamber conditions. “Time (days)” refers to the duration of drought treatment (day 0 denotes the last day of watering for treated plants). (a) Soil water content. (b) Transpiration rate. (c) Non‐photochemical quenching (NPQ) values at the end of the high light phase (HL) of fluctuating light (FL) analysis (HL NPQ). (d) Fv/Fm. (e) Fluctuating light (FL)‐induced photodamage. (f) Lifetime of qZ component of the reversible NPQ in FL analysis. (g) Amplitude of qZ component in FL analysis. Asterisks indicate the *p*‐values of the control versus drought comparison by two‐way Analysis of variance analysis (.01 < *p* < .05 is marked by *, .001 < *p* ≤ .01 by **, *p* < .001 by ***). Data are means ± SD (*n* = 5). NPQ, non‐photochemical quenching [Colour figure can be viewed at wileyonlinelibrary.com]

**FIGURE 3:**
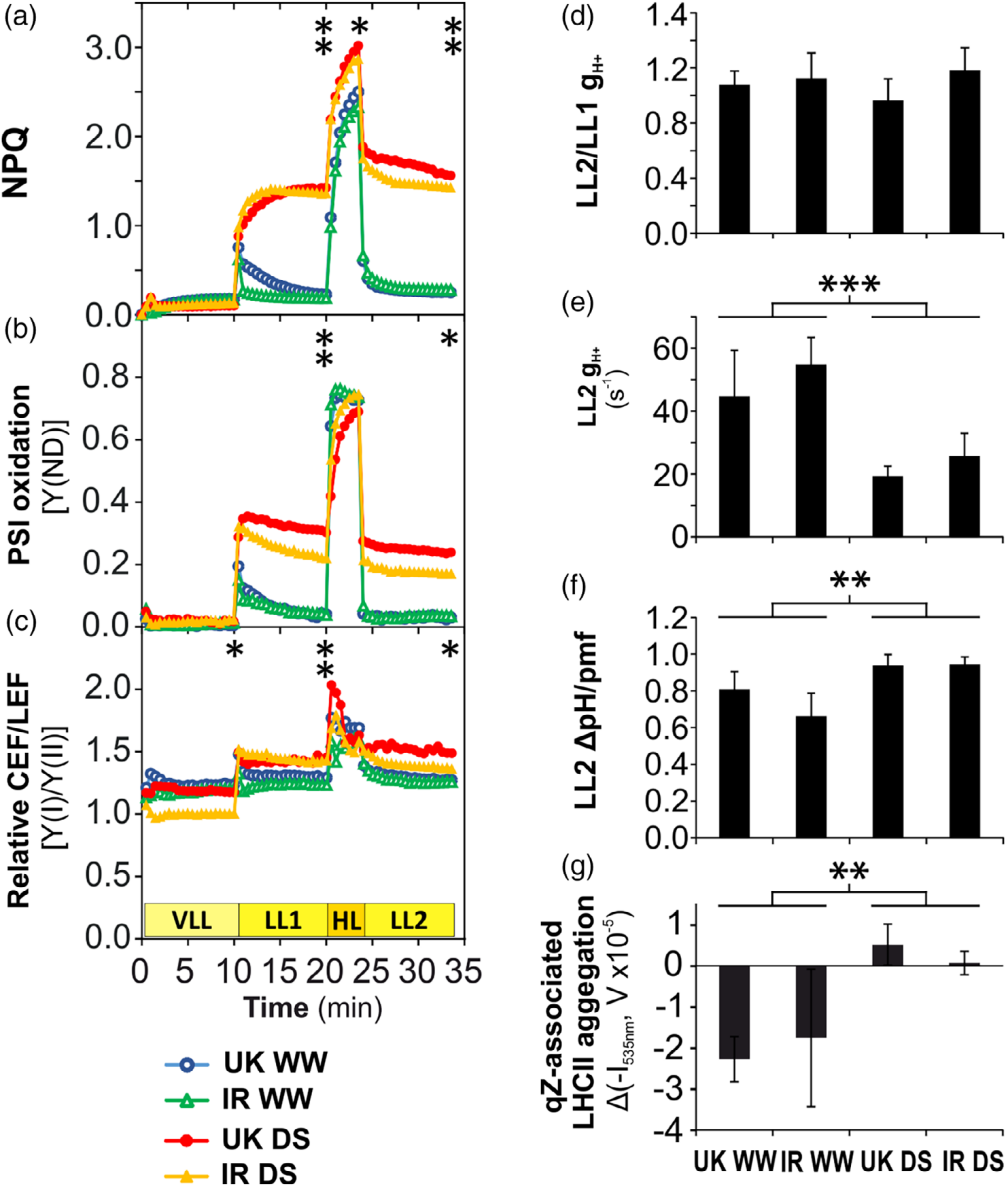
Short‐term modifications (time scale of minutes) of light reactions in the UK and IR wheat cultivars in the chamber experiment during Drought Phase III. (a‐c) Fluctuating light (FL) analysis. (a) Non‐photochemical quenching (NPQ). (b) Photosystem I (PSI) oxidation. (c) Relative changes of cyclic and linear electron flow ratio (CEF/LEF). (d) Ratio of proton conductivity through chloroplast ATP synthase (g_H+_) between LL1 and LL2 light phases. (e) g_H+_ at LL2. (f) Trans‐thylakoid ΔpH at LL2 (as fraction of proton motive force, pmf). (g) Relative variations of the aggregation of LHCII trimers associated to qZ. Asterisks indicate the *p*‐values of the control versus drought comparison by two‐way Analysis of variance analysis (.01 < *p* < .05 is marked by *, .001 < *p* ≤ .01 by **, *p* < .001 by ***) at the end of every light phase. Data are means ± SD (*n* = 3) (error bars are not indicated from panel a to c for clarity). WW, well‐watered; DS, drought‐stressed [Colour figure can be viewed at wileyonlinelibrary.com]

Measurements of NPQ shown in Figures 2 and S3c‐e were carried out by Imaging‐PAM fluorometer (Walz, Germany). Measurements presented in Figure 3 were performed by Dual‐PAM‐100 fluorometer (Walz, Germany). In both analyses, saturating pulses (SPs) were applied every 30 s (8,000 μmol photons m^−2^ s^−1^ intensity) to allow the estimation of photosynthetic parameters (Baker, 2008). Before measurements, plants were acclimated to darkness for 15 min. In the greenhouse, NPQ was measured by MultispeQ v1.0 (PhotosynQ platform, https://photosynq.org, U.S.A.) (Figures 1c and S1) according to Tietz, Hall, Cruz, and Kramer (2017). The PSII efficiency was measured as (Fm′ − F′)/Fm′ (Genty et al., 1989). The PSI efficiency was determined according to Klughammer and Schreiber (1994).

In the “FL analysis,” carried out both by Imaging‐PAM and Dual‐PAM100, leaves were illuminated for 10 min with 57 μmol photons m^−2^ s^−1^ (hereafter defined as “very low light” phase, VLL), followed by 10 min of 166 μmol photons m^−2^ s^−1^ (defined as “first low light” phase, LL1) and a subsequent “high light” phase (HL) for 3.5 min at 1466 μmol photons m^−2^ s^−1^, finally followed by a second low light (LL2) phase (at the same intensity as LL1), which was prolonged until a steady‐state NPQ value was reached (Figure S3c). The terms “low” and “high” light are defined in relation to the major growth light intensity (i.e., 450 μmol photons m^−2^ s^−1^). The light intensity of VLL had the purpose to induce a very low level of “photosynthetic control,” that is, the feedback downregulation propagated through the decrease of thylakoid lumenal pH (Bendall, 1982; Harbinson, Genty, & Foyer, 1990; Rumberg & Siggel, 1969), in such a way that NPQ and PSI oxidation were not affected by drought stress. Consequently, VLL can serve as a reference condition for evaluating the relative changes of the in vivo parameters of photosynthetic activity. The duration and intensity of the HL phase were established after pretests, with the aim of inducing maximal reversible NPQ while provoking a low level of photodamage in nonstressed plants. The PSII photodamage induced by the HL phase of FL analysis was estimated by the difference between the steady‐state NPQ value in LL2 minus NPQ at the end of LL1 and denoted as “FL‐photodamage” (Figures 2e and S3c). The NPQ decay during LL2 was fitted by a double exponential curve, allowing characterization of the two components of HL‐induced reversible NPQ: a fast component, attributable to qE, and a slow component, attributable to qZ (Figure S3c). The fitting of NPQ decay was carried out by the *nls* function in R (“stats” package) on the Imaging‐PAM data. The lifetime of qE could not be exactly determined, because the frequency of the measuring points (limited by the frequency of SPs) was too low (30 s against a lifetime range of about 20–30 s). A much higher SP frequency would alter chlorophyll fluorescence kinetics, because the SP application induces a transient perturbation of PET. The amplitudes of qE and qZ, on the contrary, could be accurately determined because of the very different order of magnitude in the lifetime of fast and slow components. The confidence limits of the fitted parameters and the p‐values associated to their estimation are indicative of optimal fitting (Figure S4).

The value of leaf absorptivity (Abs) used for calculating linear electron flow (LEF) was estimated by SPAD measurements (differential transmittance at 650 and 940 nm; SPAD‐502Plus Chlorophyll Meter, Konika Minolta, Japan), based on the equation Abs = 89.2 − 56.8*exp(−0.0723*SPAD), published by (Bauerle, Weston, Bowden, Dudley, & Toler, 2004). The mean SPAD values were 41.2, 40.9, 41.5, and 39.8 for UK WW, IR WW, UK DS, and IR DS, respectively. Because SPAD values were not significantly different between cultivars and treatments, the Abs value was fixed at 0.86 for all measurements.

Fv/Fm ratio (maximal PSII efficiency in dark acclimated leaves) was measured by PEA fluorometer (HansaTech, United Kingdom) after 30 min dark incubation.

The proton conductivity through chloroplast ATP synthase (gH+) and the proton motive force (pmf) components (ΔpH and ΔΨ) were determined by the dark‐interval relaxation kinetic of electrochromic shift (DIRK method, [Sacksteder & Kramer, 2000; Cruz, Sacksteder, Kanazawa, & Kramer, 2001]). Two signals were recorded simultaneously using a Walz Dual‐PAM 100 equipped with P515/535 module, according to the manufacturer's instruction (https://www.walz.com/downloads/manuals/dual-pam-100/515_535_module_01.pdf): the differential light transmittance at 550 and 515 nm, and the transmittance at 535 nm (I_535nm_). Plants were acclimated to darkness for 5 min in order to relax pmf, then illuminated with actinic light at the intensity of 166 μmol photons m^−2^ s^−1^ for 10 min (first low light phase, LL1). After this phase, actinic light was increased to the intensity of 1,287 μmol photons m^−2^ s^−1^ for 3.5 min (high light phase, HL). Subsequently, actinic light intensity was set again at the value of 166 μmol photons m^−2^ s^−1^ for 5 min (LL2 phase), followed by 30 s of darkness to determine the pmf, ΔpH and ΔΨ. The g_H+_ parameter was estimated at the end of LL1 and LL2. Absorbance changes at 535 nm are associated to a pool of zeaxanthin (J‐type zeaxanthin) and can be used to monitor the aggregation of LHCII trimers (Murchie & Ruban, 2020). The I_535nm_ signal during LL2 (from 900 to 1,100 s since the beginning of the whole analysis) showed a biphasic decrease: a first phase, relaxing in approximately one minute, and a second phase, showing a much slower (and approximately linear) decline (Figure S6b). The second phase of the I_535nm_ signal decay was used to monitor changes of LHCII aggregation during the qZ decay (qZ‐associated LHCII aggregation).

Gas exchange measurements, simultaneously to the acquisition of chlorophyll fluorescence parameters, were conducted by using the portable gas exchange fluorescence system GFS‐3000 (Walz, Germany). In the measuring cuvette, air CO_2_ concentration was maintained at 400 ppm, temperature at 25°C, relative humidity at 55%, flow rate at 600 μmol/s, and oxygen concentration was at ambient level.

### 2.10 Mapping wheat proteins to KO ids

We map functionally uncharacterized wheat proteins to KEGG orthologous (KO) groups (Kanehisa & Goto, 2000). Using the wheat protein as a seed, we performed a targeted orthologs search using HaMStR‐OneSeq (Jain, Roustan, Weckwerth, & Ebersberger, 2018) (https://github.com/BIONF/HaMStR) in the KEGG annotated protein sets of 30 organisms (Kanehisa & Goto, 2000). For more information see Table S1.

### 2.11 Statistical analysis

All experimental results on UK and IR cultivars were evaluated via two‐way Analysis of variance (ANOVA) analysis, carried out by the *aov* function in R (“stats” package), considering as factors “drought treatment” and “wheat genotype.” Only the significant differences attributed to the factor “drought treatment” are reported and discussed. For the sake of robustness, we consider only effects equally seen in both cultivars.

## 3 RESULTS

### 3.1 Definition of drought phases in wheat grown in greenhouse and chamber conditions

In the greenhouse, 4‐week‐old spring wheat plants were subjected to drought stress by complete water withdrawal for 24 days. After drought, plants were rewatered until harvest in order to observe the effects of water deficit to final biomass and grain yield. Soil water content was maintained at an optimal range for plant growth (around 90% field capacity) in control (well‐watered) plants (Figure 1a). At the initial phase of drought treatment, the soil water content decreased nearly linearly, indicating the availability of extractable water. At around day 16 of the treatment, the decrease slowed down and approached a plateau at day 24. The transpiration rate, monitored by measuring the daily water loss from every pot, was indicative of the water‐deficiency stress (Figure 1b). It began to decrease at day 8 of drought treatment in both cultivars. In stressed plants, NPQ (measured in relation to the photosynthetically active radiation [PAR]) started to increase at day 13 (for every condition the data from the two cultivars were jointly analyzed to increase the accuracy of the regression slope analysis) (Figures 1c and S1). The sunlight‐induced photodamage was monitored by the Fv/Fm parameter, measured after 30 min of dark incubation, which is optimal to let active NPQ relax, and to reveal differences in photodamage (Figure 1d). Although the decrease of Fv/Fm in stressed plants was numerically small (as usually occurring in slowly developing stresses), it was significant in the last 6 days of drought treatment. In Figure S2, we report the outdoor sunlight intensity illuminating the greenhouse during the drought treatment. It is important to note that this drought treatment, although did not induce a dramatic PSII damage (Fv/Fm), had remarkable effects on the final shoot biomass and grain yield (Figure 1e–g).

In summary, based on monitoring of photosynthesis and of transpiration and soil water content, we could distinguish four phases of drought acclimation in the greenhouse (Figure 1):

*Drought Phase I*: Soil water content decreased with no changes of the transpiration rate, NPQ, and PSII photodamage, as compared to the well‐watered control plants (days 1–7).

*Drought Phase II*: The transpiration rate decreased with no change of NPQ and FL‐induced photodamage, as compared to the control (days 8–12).

*Drought Phase III*: NPQ values increased with no increase of FL‐induced photodamage, as compared to the control (days 13–17).

*Drought Phase IV*: NPQ further increased and FL induced more photodamage in stressed plants, as compared to the control (days 18–24).

These phases will subsequently be used throughout the manuscript.

A similar drought treatment was applied on the same wheat cultivars in a climatic chamber under controlled ambient conditions (Figure 2). The soil water content and the transpiration rate decreased similarly in the chamber and in the greenhouse (cf. Figure 1a,b with Figure 2a,b). Leaf relative water content (RWC) reached an average value of approximately 60% in both wheat cultivars, determined at the end of drought treatment (Figure S3a). At the last day of the drought treatment, stomatal conductance was measured, revealing almost completely closed stomata at this point (Figure S3b). To investigate the adjustment of the photosynthetic activity in drought stressed wheat, the “FL analysis” was applied at different time points along the whole extent of drought treatment. This analysis partially resembles a “light curve,” a commonly used analysis monitoring the photosynthetic activity at increasing light intensities. However, the addition of the second low light phase (LL2) provided further information, such as the quantification of photodamage and NPQ relaxation kinetics (described in more details in Material and Methods). The NPQ in high light (HL NPQ) remained unaltered for several days during the drought treatment and then began to increase on day 11 in stressed plants as compared to the control (Figure 2c). NPQ showed no differences between control and stressed plants during the entire drought stress treatment in the very low light (VLL) illumination phase (Figure S3d), and it increased in LL1 from day 14 of treatment on (Figure S3e). Two parameters were used to monitor photodamage, namely, Fv/Fm and FL‐photodamage: the former reveals the effects of growth conditions, and the latter estimates the photodamage induced by the HL phase of FL‐analysis, thus giving physiological information similar to the Fv/Fm parameter measured under natural FL in the greenhouse experiment. While the Fv/Fm values were similar between well‐watered and stressed plants along the whole drought treatment (Figure 2d), FL‐photodamage was significantly higher in stressed plants in the last 2 days of the treatment (Figure 2e). Taking into account the time‐course of all parameters, the chamber experiment had similar effects in inducing NPQ and revealed the same drought phases as the greenhouse experiment under sunlight suggesting that the former can be effectively used as a proxy for field conditions.

In the chamber experiment, water deficit induced clear modifications of NPQ kinetics. In drought‐stressed plants the lifetime of qZ in LL2 significantly increased compared to control on day 14, showing two phases in both cultivars: the first phase where values increased up to around 500 s and the second phase where lifetime steeply increased beyond 1,000 s (Figure 2f). The qZ amplitude, calculated as percentage of the HL‐phase‐induced NPQ, started to increase on day 12 of treatment (Figure 2g). More information on the fitting of NPQ decay in LL2 is shown in Figure S4. Moreover, the relationship between qZ lifetime and HL NPQ in the Drought Phases III and IV was investigated (Figure S5). In well‐watered plants, qZ lifetime and HL NPQ were slightly negatively correlated, while under drought they were positively correlated and the variation of NPQ explains 25% of the qZ lifetime increase. These results suggest that the changes of NPQ kinetics were not caused merely by the enhancement of maximal NPQ.

### 3.2 Drought phase III is ideal for studying fast short‐term acclimatory mechanisms of the photosynthetic machinery

In the chamber experiment, stressed plants in Drought Phase III exhibited changes of NPQ maximal values and kinetics with no increase of photodamage compared to well‐watered plants (Figure 2). This observation clearly indicated the presence of regulatory mechanisms that are independent of photodamage. For this reason, Drought Phase III was chosen for studying more deeply the changes of NPQ in the time scale of minutes. Particularly interesting was the decrease of the NPQ decay rate upon shift from high to low light, which hereafter will be denoted as “NPQ slowdown.” To further investigate the molecular basis of NPQ slowdown, we conducted a “FL analysis” (including the same light steps described in Figure S3c) by monitoring the activities of PSII, PSI, and the chloroplast ATP synthase as well as the relative variation of LHCII aggregation in UK and IR plants in Drought Phase III in the chamber conditions. Significant differences between control and stressed plants are marked in Figure 3. However, the most useful information for investigating the NPQ slowdown stems from the comparison between LL1 and LL2 on the same leaf, rather than between control and stressed plants. In drought‐stressed plants, NPQ in LL2 tended to gradually reach the same level as in LL1 (Figure 3a), while PSI oxidation values in LL2 continued the same kinetic trend as in LL1 (Figure 3b). Because both NPQ and PSI oxidation are triggered by lumen acidification, this result suggested the existence of an additional factor, other than lumen pH, influencing NPQ kinetics. The relative values of cyclic and linear electron flow ratio (Relative CEF/LEF), measured by the ratio of PSI and PSII efficiencies, showed no significant alteration between LL1 and LL2, both in control and stress conditions (Figure 3c). The steady‐state level of the relative CEF/LEF in LL1 was significantly higher than the last value in VLL, both in control and stress conditions (*p*‐values equal to .048 and .0033, respectively; VLL‐LL1 comparison by two‐way ANOVA). However, in control, the average CEF/LEF at LL1 increased only 3.1% compared to VLL, while in stressed plants it increased 34.0% (the CEF/LEF increments from VLL to LL1 were significantly higher in stress compared to control at *p*‐value = .0015, two‐way ANOVA). Because CEF in VLL is presumably at the minimal value in all conditions, this observation indicated an increase of absolute CEF in LL1 and LL2 under drought stress compared to well‐watered condition. The values of PSII and PSI efficiencies are reported in Figure S7a,b. Similarly to CEF, the chloroplast ATP synthase conductivity showed unaltered values between LL1 and LL2 both in well‐watered and drought conditions (Figure 3d). In the LL2 phase, the ATP synthase conductivity in stressed plants was about half than in control (Figure 3e), while the lumenal ΔpH (as fraction of pmf) slightly but significantly increased in stressed plants (Figure 3f). Simultaneously to the measurement of ATP synthase conductivity and ΔpH, the relative variations of the LHCII trimers aggregation associated to qZ were monitored by the light transmittance at 535 nm (I_535nm_). Drought treatment induced a change of the variation from negative values to values around zero, revealing a slowdown of LHCII disaggregation in concomitance to qZ decay (Figure 3g).

To better characterize Drought Phase III, a light curve with the simultaneous measurement of chlorophyll fluorescence and gas exchange was performed. Stomata conductivity, LEF and net CO_2_ assimilation (A_CO2_), although severely affected by water deficit, were still responsive to the increase of light intensity (Figure S7c‐e). In the A_CO2_ versus LEF plot (Figure S7f), the regression line slopes related to the two conditions were compared in the drought‐stress range of LEF (until 85 μmol e^−^ m^−2^ s^−1^). The reduced slope of stress data (compared to well‐watered plants) indicates the increase of the portion of photosynthetic electron flow directed to alternative electron sinks (*p*‐value <2e‐16, Analysis of covariance [ANCOVA]).

**FIGURE 4:**
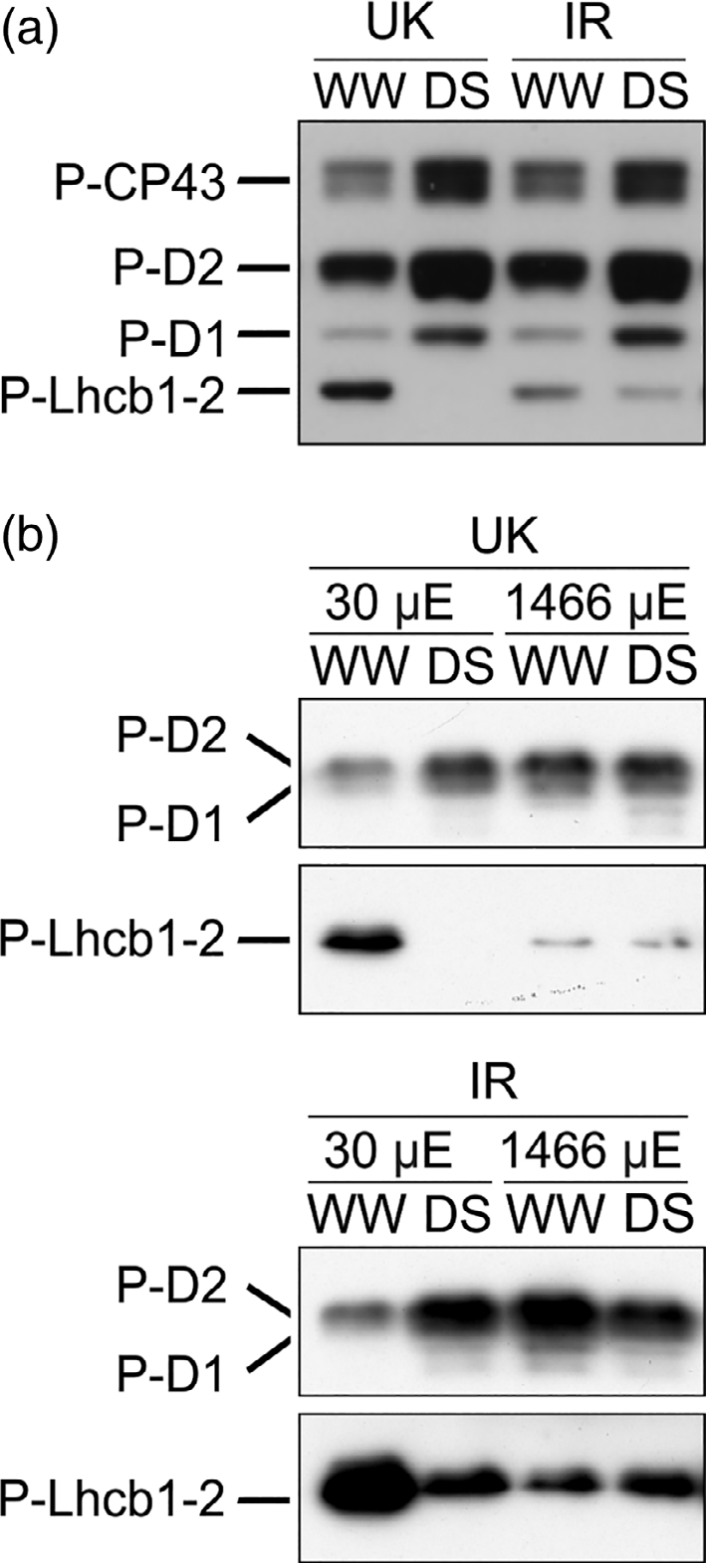
Phosphorylation of photosystem II (PSII) and Light Harvesting Complex II (LHCII), detected by anti‐phospho‐threonine immunoblotting, in the UK and IR wheat cultivars in well‐watered condition (WW) and in drought stress (DS) in the growth chamber at Drought Phase III. (a) Phosphorylation of PSII (CP43, D2, and D1 subunits) and LHCII (Lhcb1 and Lhcb2 subunits) proteins under steady‐state illumination in Drought Phase III. (b) PSII‐LHCII phosphorylation after shifting from growth light (450 μmol photons m^−2^ s^−1^) to 30 μmol photons m^−2^ s^−1^ (μE), or to 1,466 μmol photons m^−2^ s^−1^ for 10 min. P‐, phosphorylated

An important mechanism regulating the photosynthetic activity upon changes of light intensity is protein phosphorylation (Goldschmidt‐Clermont & Bassi, 2015; Grieco, Jain, Ebersberger, & Teige, 2016; Pribil, Labs, & Leister, 2014; Rochaix, 2014; Tikkanen & Aro, 2012). The levels of PSII‐LHCII phosphorylation were determined in wheat leaves harvested under steady‐state illumination (growth light at 450 μmol photons m^−2^ s^−1^) at day 14 of the chamber experiment (corresponding to Drought Phase III for the drought stressed plants). In both cultivars subjected to water deficit, the phosphorylation of D1, D2, and CP43 subunits of PSII was found to clearly increase, while LHCII (Lhcb1 and Lhcb2 proteins) phosphorylation decreased, as compared to the well‐watered control condition (Figure 4a).

Short‐term modifications of PSII‐LHCII phosphorylation upon fast changes of light intensity were also investigated. Leaves, still attached to the plant, were shifted for 10 min from growth illumination (450 μmol photons m^−2^ s^−1^) to lower (30 μmol photons m^−2^ s^−1^) or higher light intensity (1,466 μmol photons m^−2^ s^−1^) supplied by the Imaging‐PAM fluorometer. At the end of the illumination period, the illuminated leaf section was cut and immediately frozen in liquid nitrogen. In leaves exposed to high light, both well‐watered and drought‐stressed plants showed a typical response of PSII‐LHCII phosphorylation to short‐term exposure to high light (Tikkanen, Grieco, Kangasjärvi & Aro, 2010), that is, PSII was highly phosphorylated, while LHCII showed a low phosphorylation level (Figure 4b). Upon exposure to low light intensity, on the contrary, we observed a lower level of the LHCII phosphorylation and a higher extent of PSII phosphorylation in the drought stressed plants compared to the watered control. In drought‐stressed leaves, moreover, the phosphorylation patterns in low and high light were similar to each other.

### 3.3 In drought phase IV the drought‐induced regulatory mechanisms remain active and FL‐induced photodamage starts to increase

While the detailed functional analysis of Drought Phase III was useful to investigate the molecular basis of active regulatory mechanisms triggered by drought, the Drought Phase IV is of higher agricultural interest, because it includes FL‐induced photodamage with the active regulatory mechanisms still being present. Moreover, here also the connection between the fast acclimation responses in the photosynthetic machinery related to light‐harvesting and dissipation of excess energy need now also to be connected to the “use” of harvested energy by photosynthesis, for example, in the reaction of CO_2_‐fixation in the CBB cycle. To get insights into these processes as well, we collected leaves at the end of the Drought Phase IV in chamber experiments for detailed phospho‐proteomic, proteomic, and biochemical analysis.

Wheat plants in Drought Phase IV showed a similar pattern of PSII‐LHCII phosphorylation as in Drought Phase III (cf. Figure 4a with Figure S8a). In the next step, we extended our analysis beyond PSII and LHCII and analyzed general changes of thylakoid protein phosphorylation in wheat leaves in Drought Phase IV. We found that phosphorylation of CP29 protein, one of the minor antennae of PSII‐LHCII complexes, was not detectable in control plants, while CP29 showed a very low level of phosphorylation in stressed plants (Figure S8b). Therefore, we extended our analysis beyond the components of photosynthetic light harvesting by PSII and LHCII and performed an unbiased shot‐gun proteomics analysis to uncover changes in total protein abundance as well as changes in protein phosphorylation (Table S2). Here we observed a drought‐induced decrease of calcium sensing protein (CaS) phosphorylation and identified a phosphosite at the β‐subunit of chloroplast ATP synthase, which, however, was not significantly modified by water deficit.

Changes in the PSII‐LHCII phosphorylation status are associated with changes in the antenna distribution between photosystems and can therefore be detected via chlorophyll fluorescence emission spectra at the temperature of 77 K (Tikkanen et al., 2006). This analysis revealed that drought treatment induced a slight decrease of the fluorescence emitted by PSII (685 nm‐centered peak, denoted as F685) in comparison to PSI fluorescence (741 nm‐centered peak, F741) in both cultivars (F685/F741 ratio decreased 3.5% in UK and 6.2% in IR, respectively; *p*‐value = .0047 for stress vs. control comparison) (Figure S8d).

**FIGURE 5:**
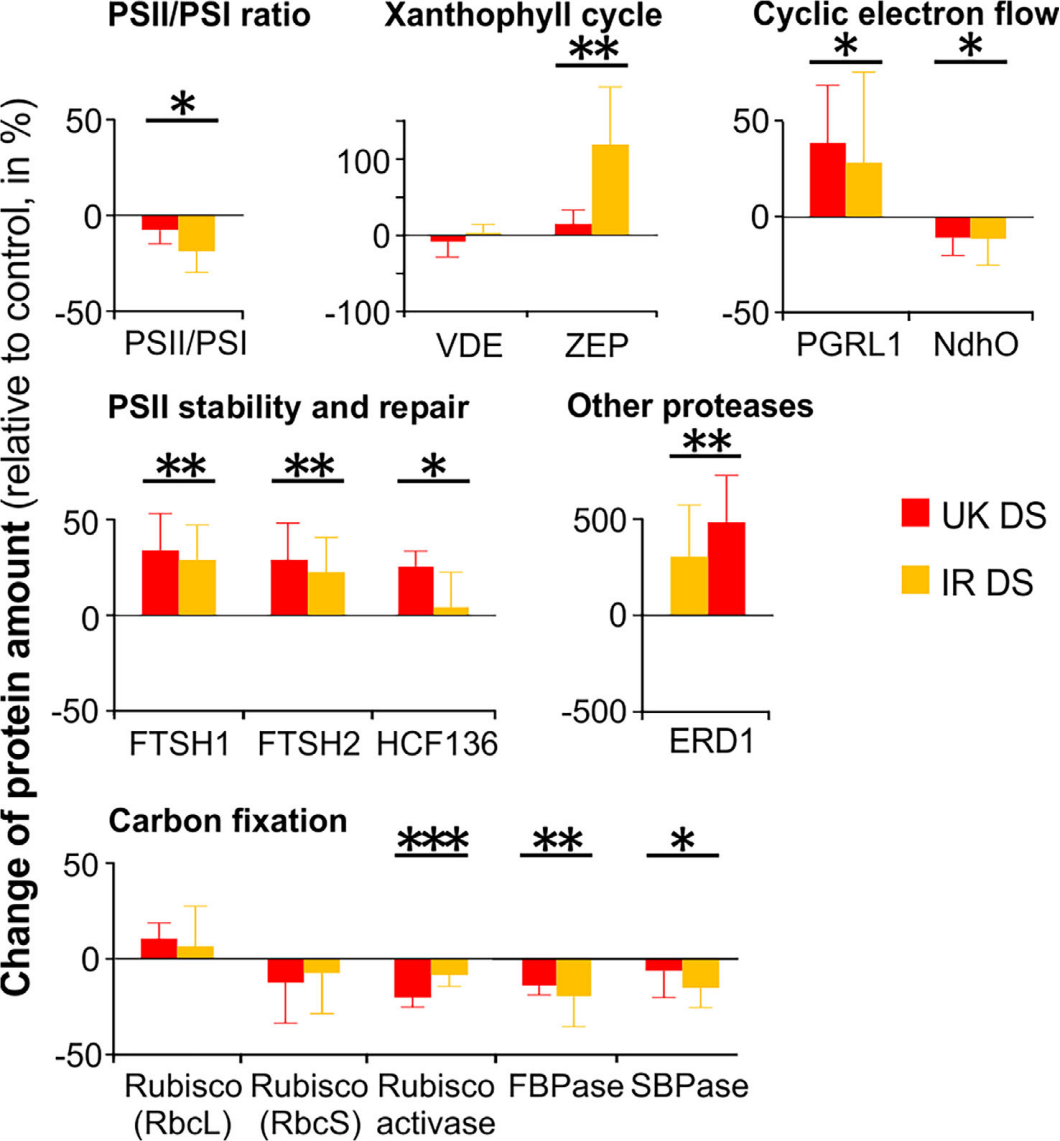
Variation of protein content under drought stress (DS), relative to the respective control (well‐watered) plants (in percentage) in UK and IR wheat cultivars in the chamber experiment at Drought Phase IV. Data are means ± SD (*n* = 5). Asterisks indicate the *p*‐values of the control versus drought comparison by two‐way Analysis of variance analysis (.01 < *p* < .05 is marked by *, .001 < *p* ≤ .01 by **, *p* < .001 by ***). More information on single proteins is available in Table S3 [Colour figure can be viewed at wileyonlinelibrary.com]

The shot‐gun proteomics identified in total 938 proteins in all 20 samples (five biological replicates per wheat variety and per condition). Only proteins detected in all samples were taken into consideration for this study (indicated as “full dataset” in Table S3). From these, 362 proteins showed significant changes in amount, of which 106 decreased and 256 increased, respectively. Totally, 98.1% of changed protein levels showed the same trend in both cultivars (Table S3, full dataset). The PSII/PSI ratio decreased slightly but significantly in both wheat cultivars under drought (decrease of 8 and 19% compared to control in UK and IR, respectively; *p*‐value = .012 for stress against control comparison, two‐way ANOVA) (Figure 5). Three proteins required for PSII stability and repair significantly increased in amount, namely, ATP‐dependent zinc metalloprotease FTSH (isoforms 1 and 2) and HCF126. Similarly, the well‐known drought stress marker gene EARLY RESPONSIVE TO DEHYDRATION 1 (ERD1), encoding a chloroplast‐targeted Clp protease regulatory subunit (Nakashima, Kiyosue, Yamaguchi‐Shinozaki, & Shinozaki, 1997), was found to be remarkably increased in both cultivars. Violaxanthin de‐epoxidase (VDE) remained unaltered, while its counterpart zeaxanthin epoxidase (ZEP) significantly increased under drought stress, if the impact of drought is analyzed on both cultivars by two‐way ANOVA. However, if analyzed separately, the two cultivars showed a different pattern, with ZEP increased in IR, but not in UK (Figure 5). No significant changes in the small and large subunits of Rubisco were detected, while in agreement with the observed reduced CO_2_ fixation rates under drought stress, a decrease in the amounts of CBB cycle enzymes was observed (Figure 5). Also here, similar reduced amounts of the involved thioredoxins were observed (Table S3). Among mobile photosynthetic electron carriers, FNR1 decreased in stressed plants. Regarding the components of CEF, the subunit O of NAD(P)H dehydrogenase complex (NdhO) decreased slightly but significantly in stressed plants. PGRL1, on the contrary, increased at 32 and 37% compared to control in UK and IR, respectively, under drought conditions. Proteins composing light‐harvesting complexes did not show any significant change in amount. Notably, neither the amounts of the Cytb6f complex or plastocyanin nor the chloroplast ATP synthase changed significantly. Considering other proteins that can induce NPQ, the PsbS protein showed no significant change. Among the components of CEF, PGRL1 increased at a similar extent in both cultivars under drought, while NdhO exhibited the opposite trend. Enzymes involved in chlorophyll biosynthesis (geranylgeranyl reductase, magnesium chelatase, protochlorophyllide oxidoreductase A) significantly decreased in stressed plants. The ratio between mitochondrial and chloroplast ATP synthase grew by 43 and 16%, respectively, in UK and IR (*p*‐value = .0014 for stress against control comparison). Also the amount of CaS increased. Finally, several components of the proteasome and ROS scavenging system significantly increased in drought‐stressed wheat plants (Table S3).

Biochemical and pigment analysis showed that the chlorophyll amount per leaf area and the chlorophyll *a*/*b* ratio (Chl *a*/*b*) did not differ between control and treated plants (Table 1). No significant variation was observed in the content of violaxanthin, antheraxanthin, and β‐carotene (Table 1). The lutein/chlorophyll *a* ratio, on the contrary, significantly increased in stressed plants as compared to the control, while zeaxanthin was not detected. While the concentration of the stress hormone ABA dramatically increased in drought as expected, the levels of MDA, a marker of lipid peroxidation, showed no differences between control and stressed plants (Table 1). This indicates that plants in water deficit could prevent long‐term damage by ROS under the growth condition despite they underwent a long period of water withdrawal.

**TABLE 1:** Biochemical analyses in two spring wheat accessions: pigment content, expressed as chlorophyll amount per leaf area, chlorophyll *a*/chlorophyll *b* ratio, and as molar ratio (mol pigment/mol chlorophyll *a*)

	Chl *a* + *b*		Violaxanthin	Antheraxanthin	Lutein	β‐Carotene	ABA	MDA
	μg/mm^2^	Chl *a*/*b*	mol/mol Chl *a*	mol/mol Chl *a*	mol/mol Chl *a*	mol/mol Chl *a*	μg/g DW	nmol/mg DW
UK WW	0.585 ± 0.053	3.936 ± 0.141	0.069 ± 0.008	0.0086 ± 0.0039	0.136 ± 0.015	0.128 ± 0.016	1.772 ± 3.188	10.763 ± 3.231
IR WW	0.525 ± 0.052	3.870 ± 0.103	0.065 ± 0.003	0.0068 ± 0.0033	0.140 ± 0.007	0.126 ± 0.010	1.986 ± 1.265	7.998 ± 1.867
UK DS	0.530 ± 0.052	3.764 ± 0.136	0.067 ± 0.005	0.0079 ± 0.0042	0.166 ± 0.024	0.130 ± 0.009	13.112 ± 6.721	9.507 ± 1.116
IR DS	0.526 ± 0.052	3.685 ± 0.170	0.062 ± 0.003	0.0063 ± 0.0040	0.149 ± 0.007	0.126 ± 0.005	26.978 ± 2.995	8.832 ± 1.016
*p*‐value	ns	ns	ns	ns	0.006	ns	5.16E‐08	ns

*Note:* The *p*‐value refers to the control versus drought comparison by two‐way Analysis of variance analysis. ns = not significant (*p* ≥ .05).

Abbreviations: ABA, abscisic acid; DW, dry weight; MDA, malondialdehyde.

## 4 DISCUSSION

### 4.1 Distinct drought phases can be defined during drought progression in wheat

In the great majority of cases, agriculture is performed on soil types that can retain high amounts of water. Consequently, in case of lacking rain or irrigation, a field usually dries out in the time scale of weeks (Boonjung & Fukai, 1996; Lilley & Fukai, 1994). During such slow soil dehydration, the plant has sufficient time to implement morphological and molecular modifications in order to react to water deficit. Nevertheless, in many previous studies, considerable loss of shoot water content was reached in a few days (often less than 7 days) (Agrawal et al., 2016; Bonhomme, Valot, Tardieu, & Zivy, 2012; Liu et al., 2009). In these conditions, a brief drought treatment corresponds to a very severe drought, as the plant does not have enough time to properly react to water deficit, as it would do on the field. In some cases, a strong response was obtained in just 1 day by immerging roots in polyethylene glycol (PEG) (Agrawal et al., 2016; Liu et al., 2009). These approaches have contributed to emphasize the damage of the photosynthetic apparatus as consequence to water deficit. In contrast, here we pursued an approach that allowed us to monitor the molecular effects of both drought and changing light conditions with a higher resolution before damage under growth light occurs. We recognized well‐defined drought phases, which were observed in growth chamber as well as in greenhouse experiments (Figures 1 and 2). In both experimental conditions, we observed a drought‐dependent rise of NPQ, confirming previous publications (e.g., Zivcak et al., 2013). However, such increase appears to be delayed compared to the drought stress measured as water loss in soil. To investigate which factors are involved in the NPQ response, we employed an indoor experimental set‐up which resembles closely the kinetic observed in the greenhouse, and therefore can be taken as a proxy of the adaptation of the plants in the field.

On this basis, we could gain insight on how long‐term structural changes of the photosynthetic machinery (time scale of days‐weeks) are related to the capacity to acclimate to water deficit in both constant illumination and fast fluctuations of light intensity (time scale of minutes). In particular, our approach allowed us to discover two novel regulatory mechanisms for the acclimation of the photosynthetic machinery to drought in wheat: (a) the acquisition of a drought‐specific configuration of the photosynthetic machinery, consisting of changes in protein stoichiometry and PSII‐LHCII phosphorylation (active under growth light); (b) the slowdown of NPQ decay upon shift from high to low light (active under FL).

### 4.2 The drought‐specific pattern of PSII‐LHCII phosphorylation is part of a new configuration that adjusts the photosynthetic machinery in long‐term

So far, only a few studies have been conducted on the role of PSII‐LHCII phosphorylation in drought. Prolonged water deficit in Arabidopsis induced a decrease of PSII phosphorylation (precisely of D1 protein) but did not change LHCII phosphorylation (Chen et al., 2016). Pea plants (*Pisum sativum*), subjected to severe drought by water withdrawal showed an increase of phosphorylation in both PSII and LHCII (Giardi et al., 1996). In barley (*Hordeum vulgare* L.), on the contrary, Liu and coworkers (Liu et al., 2009) observed a decrease of both PSII and LHCII phosphorylation with a concomitant increase of CP29 phosphorylation, upon PEG treatment. PEG treatment induced an increase of CP29 phosphorylation in barley and maize, but not in spinach (Chen et al., 2009). In the same study, barley and maize plants showed increased CP29 phosphorylation also under high light, cold, and salt stress. The increase of CP29 phosphorylation upon exposure to high light was further observed both in dicots and monocots and associated with PSII damage (Betterle, Ballottari, Baginsky, & Bassi, 2015; Fristedt & Vener, 2011). As a result, no common pattern of PSII‐LHCII phosphorylation in drought stress can be deduced among different species. However, a remarkable difference between drought application by gradual water withdrawal and PEG treatment is that the latter induces rapid shoot dehydration together with a large decrease of PSII intactness (Fv/Fm parameter). Therefore, CP29 phosphorylation is probably a general response associated to severe PSII damage in many species, and it was proposed to favor the disassembly of PSII‐LHCII complexes in monocots exposed to abiotic stresses (Chen, Zhao, Zhang, Zeng, & Yuan, 2013). Accordingly, in the present study, CP29 phosphorylation was very low in wheat plants subjected to a drought stress that did not induce an increase of PSII damage (Figures 2 and S8).

**FIGURE 6:**
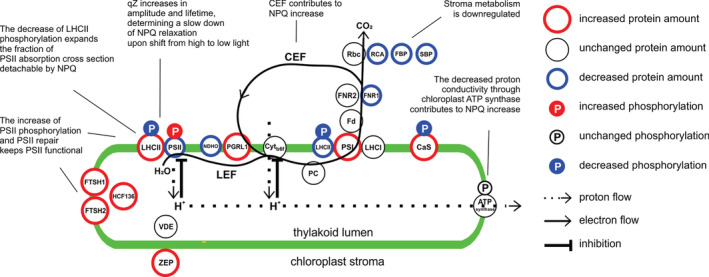
Drought‐specific configuration of the photosynthetic machinery acquired by wheat in Drought Phase IV. The figure shows changes in single proteins or protein complexes amount, relative to well‐watered condition. There are two special cases: i) light‐harvesting complex II (LHCII), which is shown in two pools, one bound to PSII, one to PSI; ii) decrease of PSII/PSI ratio is depicted as a relative change of PSII and PSI. Circles position does not represent the precise protein/complex localization. CaS, calcium‐sensing protein; Cyt b6f, cytochrome b6f; Fd, ferredoxin; FNR, Ferredoxin‐NADP reductase; FTSH, FtsH protease; HCF136, Photosystem II stability/assembly factor HCF136; LHCI, light‐harvesting complex I; LHCII, light‐harvesting complex II; NDHO, NAD(P)H:plastoquinone dehydrogenase complex subunit O; PC, plastocyanin; PGRL1, PGR5‐like protein 1; PSI, photosystem I; PSII, photosystem II; Rbc, Rubisco; RCA, Rubisco activase; VDE, violaxanthin de‐epoxidase; ZEP, zeaxanthin epoxidase. Comments summarize the drought‐induced functional modifications. LEF, linear electron flow; CEF, cyclic electron flow [Colour figure can be viewed at wileyonlinelibrary.com]

In our study, a similar drought‐induced PSII‐LHCII phosphorylation pattern was observed in Drought Phase III and IV under growth light (Figures 4 and S8). Moreover, in contrast to well‐watered plants, under drought this pattern remained similar after a short exposure to high and low light intensities. This suggests that the phosphorylation level does not change in drought stressed plants upon changes in light intensity (Figure 4). Therefore, the modification of PSII‐LHCII phosphorylation is a long‐term acclimation process for drought conditions. The physiological implications of the drought‐specific PSII‐LHCII phosphorylation pattern are better understood if several stoichiometric changes are also taken into account. All these modifications together constitute a re‐configuration of the photosynthetic machinery (schematically summarized in Figure 6). Here, the term “re‐configuration” refers to the fine‐tuning of structural changes of the photosynthetic machinery, which constitute the basis for functional modifications.

LHCII is a stable antenna of both PSII and PSI (Grieco, Suorsa, Jajoo, Tikkanen, & Aro, 2015; Grieco, Tikkanen, Paakkarinen, Kangasjärvi, & Aro, 2012; Tikkanen et al., 2006; Wientjes, van Amerongen, & Croce, 2013). The role of LHCII phosphorylation is to fine‐tune the relative distribution of LHCII between PSII and PSI (Tikkanen et al., 2006). In our drought condition, the total LHCII amount does not change compared to the control. Hence, a decrease of LHCII phosphorylation in drought implies that a higher portion of LHCII is energetically connected to PSII instead of PSI (Figure 4). This change would tend to increase the energy flow to PSII, which would result in an increased PSII peak in 77 K emission spectra. On the other hand, the decrease of PSII/PSI ratio would tend to decrease the relative amount of energy captured by PSII, which would imply a decrease of the PSII peak in 77 K spectra. However, the 77 K spectra recorded from drought‐stressed wheat were almost not altered in comparison to the well‐watered condition (Figure S8). A possible explanation is that in stressed plants the energetic imbalance that could derive from the decrease of LHCII phosphorylation is counterbalanced by the decrease of the PSII/PSI ratio (Figure 5). Because LHCII is detachable from PSII, the low steady‐state level of LHCII phosphorylation in drought‐stressed wheat gives the advantage to increase the dynamic range of energy capture, allowing high efficiency of light harvesting under low light (low NPQ) and a larger decrease of PSII antenna size (higher NPQ) under higher light intensities. In other words, in drought‐stressed wheat, the fraction of PSII absorption cross section that is detachable by NPQ is expanded. The ultimate effect is to enhance the functional flexibility of light reactions, that is, the capability to acquire high efficiency in low light and low efficiency in high light (Figures 2, 3 and S3).

At the same time, the maintenance of a high level of PSII phosphorylation favors a continuously accelerated PSII repair cycle regardless of light intensity (Figure 4). Accordingly, the PSII repair machinery and the ROS scavenging system are also upregulated, thus contributing to prevent long‐term damage of the photosynthetic apparatus (Figure 5 and Table S3). Other factors that can possibly contribute to prevent long‐term damage are lutein and the CaS protein. Lutein contributes to light‐harvesting and structural stabilization of antennae proteins (Jahns & Holzwarth, 2012), and it can quench triplet chlorophyll (Dall'Osto et al., 2006). The overexpression of CaS was related to decreased membrane damage under drought stress (Zhao, Xu, Wei, & Liu, 2015), and it could possibly contribute to the enhancement of CEF/LEF ratio (Terashima et al., 2012). In our drought condition, the overall amount of light reactions protein complexes remained basically stable (confirmed by unaltered chlorophyll content), while several components of carbon metabolism were downregulated, concomitantly to a decreased CO_2_ uptake. In parallel, the relative amount of mitochondrial proteins increased (Table S3), indicating that already at this stage of drought part of the reducing power produced by light reactions was probably dissipated by mitochondria, as suggested by previous studies (Atkin & Macherel, 2009). All these observations indicate that, under steady‐state illumination, the integrity and functionality of the photosynthetic machinery were actively preserved: light reactions were adjusted to the downregulated carbon metabolism by a change in configuration, rather than by an overall stoichiometric decrease or by protein complexes degradation (Figure 6).

The functional modifications described above were successful in preventing excessive photodamage under FL in Drought Phase III. In Drought Phase IV, instead, water deficit provoked an increase of PSII damage upon exposure to high light. In our view, the photosynthetic machinery tends to be preserved in the long‐term, while short‐term PSII damage is useful for preventing excessive production of reducing power that would damage the whole cell. The regulation of PSII photoinhibition was proposed to be the ultimate regulator of PET and to constitute a photoprotective mechanism against photodamage in PSI and excessive formation of ROS (Tikkanen, Mekala, & Aro, 2014). The decreased amount of chlorophyll biosynthesis enzymes and the increase of proteasome components indicate that long‐term degradation processes were already triggered at the end of our drought treatment, although they did not yet affect the integrity of the photosynthetic apparatus.

### 4.3 The slowdown of NPQ decay adjusts the photosynthetic activity under fluctuating light in drought‐stressed wheat

Although wheat plants were not grown under FL in the chamber condition, under drought they modified the fast NPQ kinetics by increasing qZ amplitude and lifetime, when the leaf was monitored in the FL analysis (Figures 2 and 3). This implies the presence of a long‐term modification of the photosynthetic machinery that is induced by drought and has effects on the regulation of the photosynthetic activity under fast FL (time scale of minutes). A possible explanation could rely on VDE and/or ZEP, the two antagonistic enzymes controlling zeaxanthin content. The amount of VDE did not respond to water deficit and that of ZEP, the enzyme that converts zeaxanthin to violaxanthin, did not decline in either of the cultivars. Consequently, the NPQ slowdown could be caused by changes of the enzymatic activity, rather than stoichiometry. Future studies could possibly verify if a posttranslational modification of ZEP and/or VDE occurs under drought, as a mechanism allowing a tight regulation of the enzymatic activity upon fast changes of light intensity. Because ZEP activity is a limiting step in ABA synthesis, which is important for response to water deficit, it could be advantageous to avoid decreasing the amount of ZEP in a long term. In fact, ABA increased more in IR than in UK, with IR showing a strong increase of ZEP amount (Table 1 and Figure 5). In addition, during the qZ decay in LL2 light phase, drought was likely to induce the slowdown of the disassembly of LHCII aggregates, a photoprotective (quenching) form of LHCII antennae (Figure 3g). Indeed, zeaxanthin has been shown to promote LHCII aggregation (Johnson et al., 2011), while lutein was identified as the quencher molecule in LHCII aggregates (Ruban et al., 2007). Therefore, both the kinetics of LHCII aggregation and lutein accumulation possibly jointly contribute to the slowdown of NPQ decay in drought‐stressed wheat plants.

We investigated other mechanisms that could influence ZEP/VDE activity or directly NPQ kinetics. We show that CEF and chloroplast ATP synthase activities are concurrent in increasing NPQ in LL1 and LL2 light phases under drought. The enhancement of CEF and chloroplast ATP synthase resistance to proton flux was reported in previous studies done in other species (Golding, Finazzi, & Johnson, 2004; Golding & Johnson, 2003; Huang, Yang, Zhang, Zhang, & Cao, 2012; Kohzuma et al., 2009; Tezara, Mitchell, Driscoll, & Lawlor, 1999). In our drought condition, the upregulation of CEF/LEF ratio was supported by the increased amount of PSI (relative to PSII) and of PGRL1, a component of CEF (Figure 5). The enhancement of the PGRL1‐dependent CEF in drought seems to occur at the expenses of the NDH‐dependent CEF (Figure 5). It is worth to note that, at the best of our knowledge, this study is the first one showing a decrease of chloroplast ATP synthase conductivity with no enzyme damage or stoichiometric change in drought conditions. A possible trigger of the downregulation of ATP synthase activity could be the accumulation of CBB intermediates, in particular a possible accumulation of ATP with corresponding depletion of ADP, or the depletion of stromal phosphorus (Takizawa, Kanazawa, & Kramer, 2008). Allosteric regulation or posttranslational protein modification modulating the enzymatic activity should be also considered. The phosphorylation of ATP synthase beta‐subunit at Ser496 seems not to be involved in this regulation, at least not at the drought conditions applied in this study (Table S2). However, both CEF and ATP synthase activities in LL2 did not differ from LL1 in drought‐stressed plants. This indicates that these two mechanisms do not contribute to NPQ slowdown (Figure 3).

The physiological advantage of the NPQ slowdown is most likely the possibility for the stroma metabolism to get rid of the excess of reducing power accumulated during the HL phase of FL. It is still unclear how NPQ dynamics can play a role under stress and how this correlates to grain yield. Under drought, both the amplitude and lifetime of qZ were progressively increasing when the reservoir of extractable soil water was reaching its minimum. This feature shows the potentiality for developing parameters to be applied to large‐scale phenotyping. A possibility is the screening of wheat populations by the application of FL to test the ability to recover the photosynthetic activity from high to low light. In addition, the correlation between PSII‐LHCII phosphorylation patterns and drought resistance could be also explored in large scale. This approach would open up new possibilities for crop breeding.

## 5 CONCLUSION

Here, we present a specific model for the acclimation and regulation of photosynthesis to slowly increasing levels of drought stress in wheat. We describe the specific strategy of wheat to adjust the photosynthetic machinery to drought by employing mechanisms that are independent of drought‐induced damage. This strategy consists of the fine tuning of the stoichiometry of several enzymes, together with a long‐term modification of PSII‐LHCII phosphorylation. These structural changes are associated to modifications of NPQ kinetics, which could represent an adaptive mechanism adjusting the photosynthetic activity upon FL under drought conditions. This constitutes a major difference to most previous studies, which have focused on rather harsh stress conditions and accordingly emphasized photodamage as the only cause of the downregulation of photosynthetic activity in drought. Moreover, our findings offer the basis for developing traits to be applied on crop phenotyping and future breeding programs.

## CONFLICT OF INTEREST

The authors declare no conflicts of interest.

## AUTHOR CONTRIBUTION

Michele Grieco conceived the study; Michele Grieco and Markus Teige wrote the article; Michele Grieco, Valentin Roustan, Georgi Dermendjiev, Sanna Rantala, Manuela Leonardelli, Vitus Berger, and Doris Engelmeier performed experiments; Michele Grieco, Valentin Roustan, Arpit Jain, Kerstin Neumann, Gert Bachmann, Ingo Ebersberger, Eva‐Mari Aro, Wolfram Weckwerth, and Markus Teige analyzed data and revised the manuscript.

## Supporting information


**Appendix** **S1**. Supporting information.

**Figure S1.** NPQ plotted against the incident sunlight photosynthetically active radiation (PAR), measured on well‐watered (WW) and drought‐stressed (DS) wheat plants in the greenhouse experiment.

**Figure S2.** Outdoor sunlight intensity during the greenhouse experiment described in Figure 1.

**Figure S3.** Additional data of the chamber experiment described in Figure 2.

**Figure S4.** Assessment of the quality of the NPQ decay fitting in FL analysis on UK and IR wheat cultivars (described in Figure S3c).

**Figure S5.** Linear regression analysis of qZ lifetime versus high light‐induced NPQ (HL NPQ) in the UK and IR wheat cultivars in Drought Phases III and IV of the chamber experiment, based on the data shown in Figure 2. WW = well‐watered; DS = drought stressed.

**Figure S6.** Example of raw data for the determination of the parameters described in Figure 3d‐g.

**Figure S7.** Additional data on short‐term changes of photosynthetic activity in UK and IR wheat cultivars in the chamber experiment in Drought Phase III.

**Figure S8.** Thylakoid protein phosphorylation and 77 K fluorescence emission spectra in UK and IR wheat cultivars in the Drought Phase IV of the chamber experiment.

**Table S1.** List of model organisms obtained from KEGG (Kanehisa & Goto, 2000).

**Table S2.** Phosphorylated proteins detected by mass spectrometry in the United Kingdom (UK) and Iran (IR) wheat cultivars.

**Table S3** Proteomics analysis on the United Kingdom (UK) and Iran (IR) wheat cultivars.
