# Supplementary material for: Adjustment of photosynthetic activity to drought and fluctuating light in wheat
Source: Plant Cell Environ. 2020 Mar 27;43(6):1484–500. doi: 10.1111/pce.13756 (PMC7384038; doi:10.1111/pce.13756)
Supplement: Supplementary file 1 — Appendix S1. Supporting information. [file PCE-43-1484-s001.docx]

**Supplemental text**

**Mass spectrometry protocol**

Protein extraction: Total protein was extracted from wheat leaf extract with an adapted protocol from (Roustan et al., 2017). In detail, protein were extracted with 2 ml of extraction buffer (100mM Tris–HCl pH 8.0; 5% SDS, 10% glycerol; 1.4M sucrose; 10mM DTT; supplemented with protease and phosphatase inhibitor cocktails as indicated by the supplier (Roche, Cat. No. 05 892 791 001 and Cat. No. 04 906 837 001) and samples were incubated at room temperature for 5 min with periodic vortexing. Immediately after the extraction, 1 ml Roti-phenol was added to samples, which were then vortexed and incubated for 5 min before centrifugation at 21,000 ×g for 5 min at room temperature. The supernatant was carefully transferred to a new tube. Phenol extraction was repeated a second time with 500 µl of Roti-phenol. Phenol fractions were pooled together and counter extracted with 1 ml of extraction buffer and centrifuged at 21,000 ×g for 5 min at room temperature. The supernatant was carefully transferred to a new tube. Protein precipitation was performed by mixing the phenol fraction with 2.5 volumes of 0.1M ammonium acetate in methanol. After a 16 h incubation period at −20 °C, the samples were centrifuged for 5 min at 5000 ×g. The protein pellets were washed twice with 0.1M ammonium acetate, one time with acetone and air dried at room temperature. Protein pellets were dissolved in 8 M urea/100 mM ammonium bicarbonate (AmBic) supplemented with protease and phosphatase inhibitor cocktails as indicated by the supplier (Roche, Cat. No. 05 892 791 001 and Cat. No. 04 906 837 001). Protein concentration was determined using the Bio-Rad Bradford Assay with BSA as a standard. 100 μg of total protein per sample was first reduced with dithiothreitol (DTT) at a concentration of 5 mM at 37 °C for 45 min. Cysteine residues were alkylated with 10 mM iodoacetamide (IAA) in darkness at room temperature (RT) for 60 min. Alkylation was stopped by increasing DTT concentration to 10 mM and incubating the samples in the dark at RT for 15 min. Then the urea concentration was diluted to 2 M with 50 mM AmBic/10 % acetonitrile (ACN). CaCl2 was added to a final concentration of 2 mM. Trypsin digestion (Poroszyme immobilized trypsin; 5:100 v:w) was performed at 37 °C overnight. Protein digests were desalted with C18 solid phase extraction (SPE) (Agilent Technologies, Santa Clara, USA) and carbon graphite SPE as described by (Furuhashi et al., 2014)(Furuhashi, Nukarinen, Ota & Weckwerth 2014)(Furuhashi, Nukarinen, Ota & Weckwerth 2014). After both SPEs, the corresponding eluates were pooled and dried in a vacuum concentrator.

LC-MS for proteomics: Peptide pellets were solved in 10 µL of 5% (v/v) ACN, 0.5% (v/v) formic acid (FA). The equivalent of 0.5 µg total protein was loaded on an EASY-Spray PepMap RSLC 75 μm × 50 cm column (Thermo Fisher Scientific Inc., Waltham, USA) for peptides separation. Peptides were eluted using a 240 min linear gradient from 2 to 40 % of mobile phase B (mobile phase A: 0.1 % [v/v] formic acid (FA) in water; mobile phase B: 0.1 % [v/v] FA in 90 % [v/v] ACN) with 300 nL/min flow rate generated with an UltiMate 3000 RSLCnano system. Peptides were measured with an LTQ-Orbitrap Elite (Thermo) using the following mass analyzer settings: ion transfer capillary temperature 275°C, full scan range 350-1800 m/z, FTMS resolution 120000. Each FTMS full scan was followed by up to ten data dependent (DDA) CID tandem mass spectra (MS/MS spectra) in the linear triple quadrupole (LTQ) mass analyzer. Dynamic exclusion was enabled using list size 500 m/z values with exclusion width ±10 ppm for 60 s. Charge state screening was enabled and unassigned and +1 charged ions were excluded from MS/MS acquisitions. For injection control automatic gain control (AGC) for full scan acquisition in the Orbitrap was set to 5 x 105 ion population, the maximum injection time (max IT) was set to 200ms. Orbitrap online calibration using internal lock mass calibration on m/z 371.10123 from polydimethylcyclosiloxane was used. Multistage activation was enabled with neural losses of 24.49, 32.66, 48.999, 97.97, 195.94, and 293.91 Da for the 10 most intense precursor ions. Prediction of ion injection time was enabled and the trap was set to gather 5 x 103 ions for up to 50 ms.

Data analysis: Wheat UniProt database was upload to MaxQuant 1.5 (<http://www.maxquant.org>) and Andromeda search algorithm was used (Cox and Mann, 2008; Cox et al., 2011) for peptide identification. Protein quantification was based on ion precursor intensities. Peptides identification was performed using the following settings: mass tolerance for precursor was set to 5 ppm and for fragment masses up to 0.8 Da. The maximum FDR was set to 0.01%. Two missed cleavages were allowed. The dynamic modifications allowed were: methionine oxidation (M) and protein N-terminal acetylation. The fixed modification allowed was: Carbamidomethyl (C). Further data processing was carried out with the Perseus 1.5 software (Tyanova et al., 2016). Additionally, proteins were accounted for quantification only if they were present in all samples. Perseus 1.5 software was also used for data analysis. Label-free quantification (LFQ) intensities of proteins are derived from measured MS1 intensities of at least two peptides per protein. For every sample, each relative protein amount was normalized to the sum of the LFQ intensities. In order to estimate relative changes of PSII/PSI ratio and the ratio between mitochondrial and chloroplast ATP synthase, the relative amount of every protein complex was calculated as the sum of LFQ intensities of all detected proteins composing the complex. The mass spectrometry proteomics data have been deposited to the ProteomeXchange Consortium via the PRIDE (Vizcano et al., 2016) partner repository with the dataset identifier PXD011110.

**REFERENCES**

Cox J, Mann M (2008) MaxQuant enables high peptide identification rates, individualized p.p.b.-range mass accuracies and proteome-wide protein quantification. Nature Biotechnology 26: 1367-1372

Cox J, Neuhauser N, Michalski A, Scheltema RA, Olsen JV, Mann M (2011) Andromeda: a peptide search engine integrated into the MaxQuant environment. Journal of Proteome Research 10: 1794-1805

Finn RD, Mistry J, Tate J, Coggill P, Heger A, Pollington JE, Gavin OL, Gunasekaran P, Ceric G, Forslund K, Holm L, Sonnhammer ELL, Eddy SR, Bateman A (2010) The Pfam protein families database. Nucleic Acids Research 38: D211-222

Furuhashi T, Nukarinen E, Ota S, Weckwerth W (2014) Boron nitride as desalting material in combination with phosphopeptide enrichment in shotgun proteomics. Analytical Biochemistry 452: 16-18

Jain A, Roustan V, Weckwerth W, Ebersberger I (2018) Studying AMPK in an Evolutionary Context. Methods Mol Biol 1732: 111-142

Kanehisa M, Goto S (2000) KEGG: kyoto encyclopedia of genes and genomes. Nucleic Acids Res 28: 27-30

Koestler T, von Haeseler A, Ebersberger I (2010) FACT: functional annotation transfer between proteins with similar feature architectures. BMC Bioinformatics 11: 417

Letunic I, Doerks T, Bork P (2015) SMART: recent updates, new developments and status in 2015. Nucleic Acids Research 43: D257-260

Roustan V, Bakhtiari S, Roustan P-J, Weckwerth W (2017) Quantitative in vivo phosphoproteomics reveals reversible signaling processes during nitrogen starvation and recovery in the biofuel model organismChlamydomonas reinhardtii. Biotechnology for Biofuels 10: 280

Tyanova S, Temu T, Sinitcyn P, Carlson A, Hein MY, Geiger T, Mann M, Cox J (2016) The Perseus computational platform for comprehensive analysis of (prote)omics data. Nature Methods 13: 731-740

Vizcano JA, Csordas A, Del-Toro N, Dianes JA, Griss J, Lavidas I, Mayer G, Perez-Riverol Y, Reisinger F, Ternent T, Xu Q-W, Wang R, Hermjakob H (2016) 2016 update of the PRIDE database and its related tools. Nucleic Acids Res 44: D447--456.
