# Supplementary figures and images for: Adjustment of photosynthetic activity to drought and fluctuating light in wheat
Source: Plant Cell Environ. 2020 Mar 27;43(6):1484–500. doi: 10.1111/pce.13756 (PMC7384038; doi:10.1111/pce.13756)

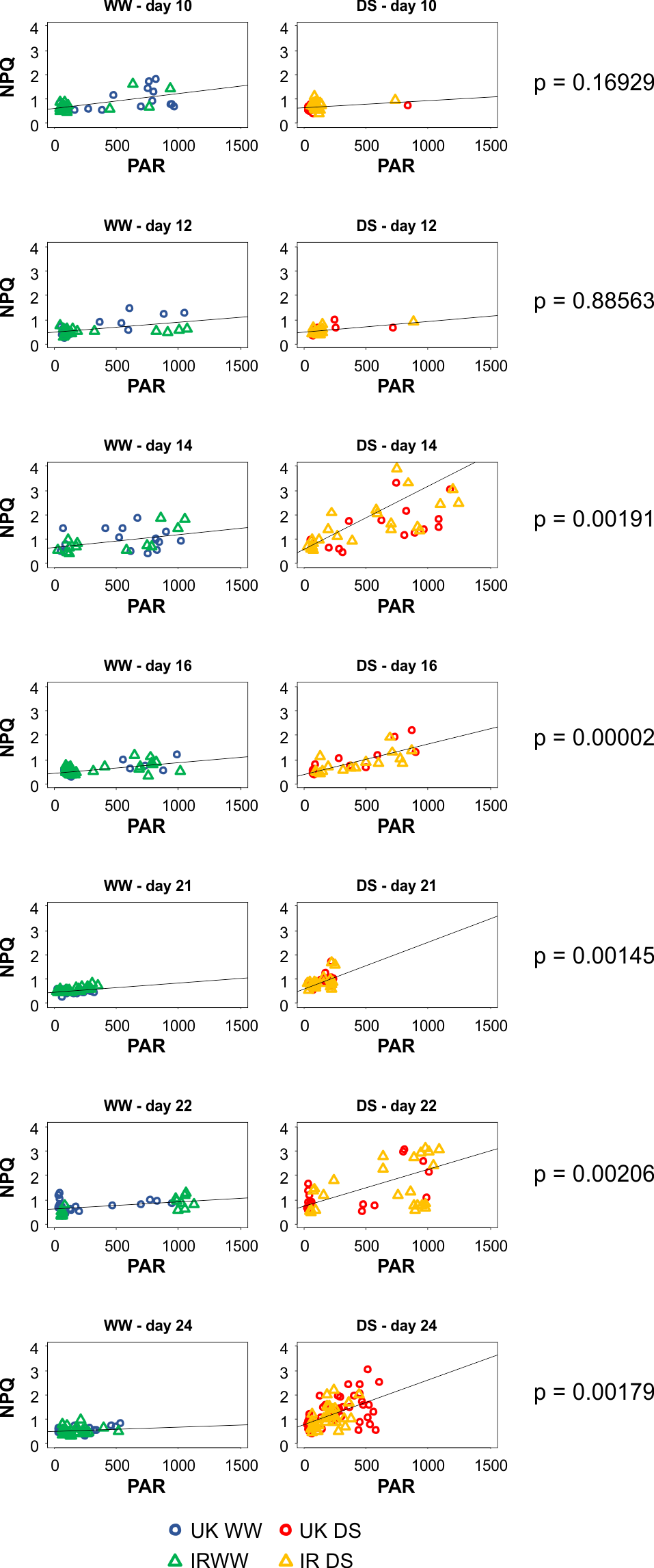

Supplement: Supplementary file 2 — Figure S1. NPQ plotted against the incident sunlight photosynthetically active radiation (PAR), measured on well‐watered (WW) and drought‐stressed (DS) wheat plants in the greenhouse experiment. [file PCE-43-1484-s002.tif]

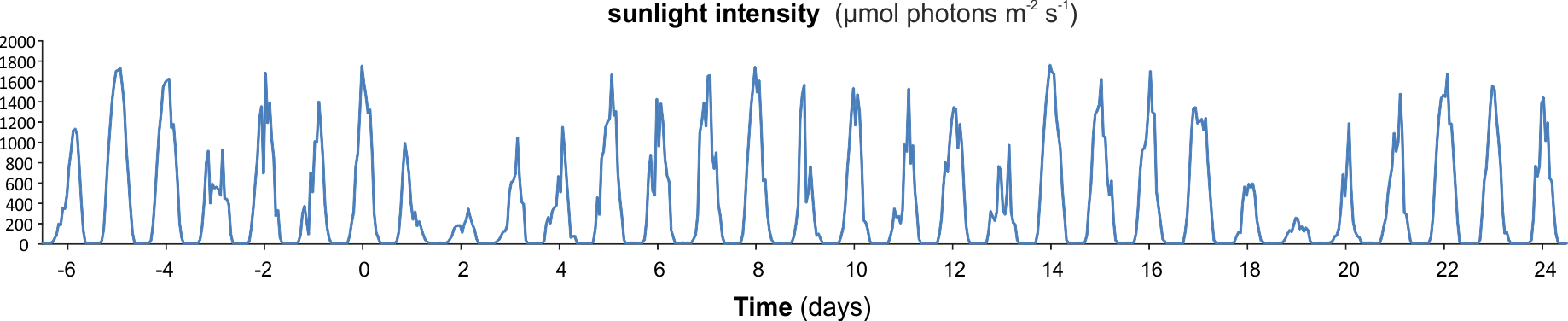

Supplement: Supplementary file 3 — Figure S2. Outdoor sunlight intensity during the greenhouse experiment described in Figure 1. [file PCE-43-1484-s003.tif]

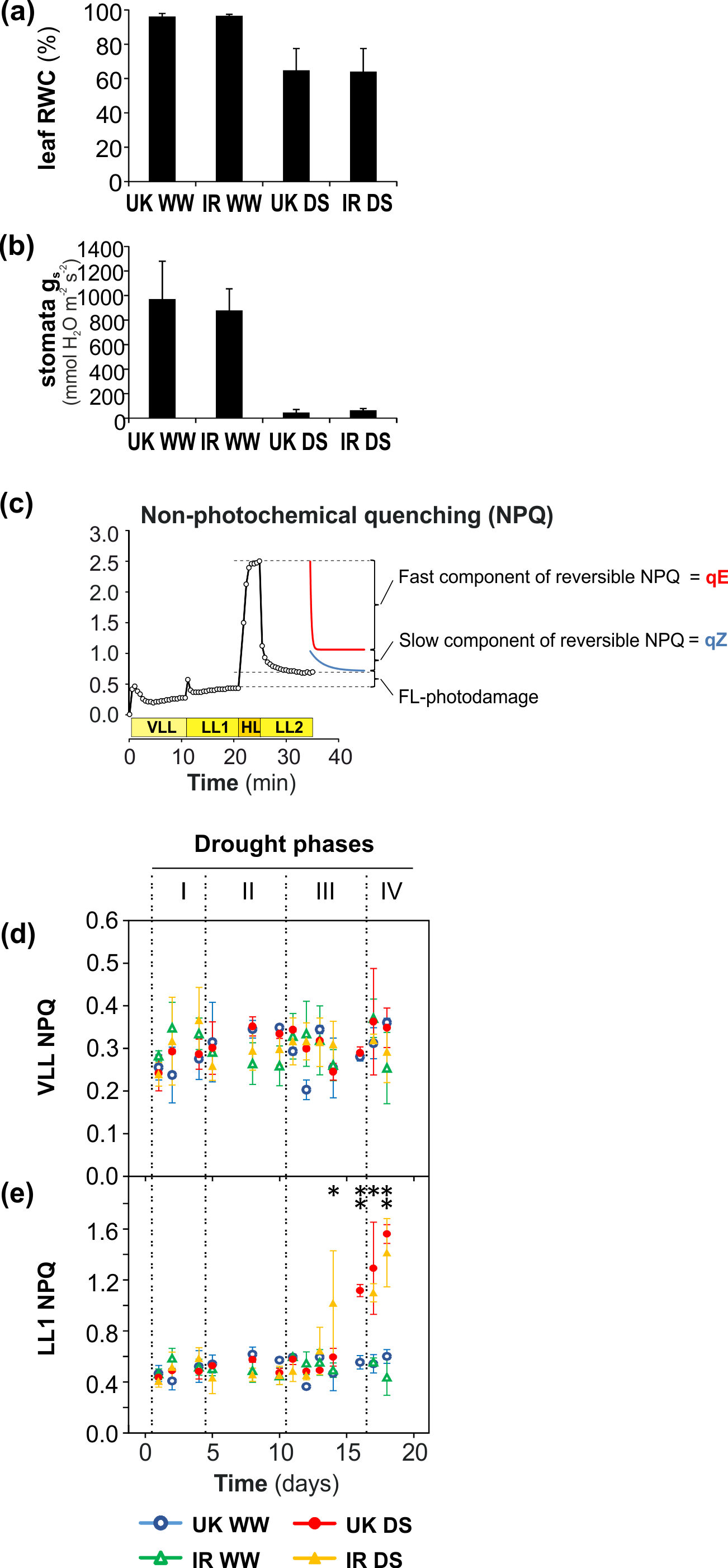

Supplement: Supplementary file 4 — Figure S3. Additional data of the chamber experiment described in Figure 2. [file PCE-43-1484-s004.tif]

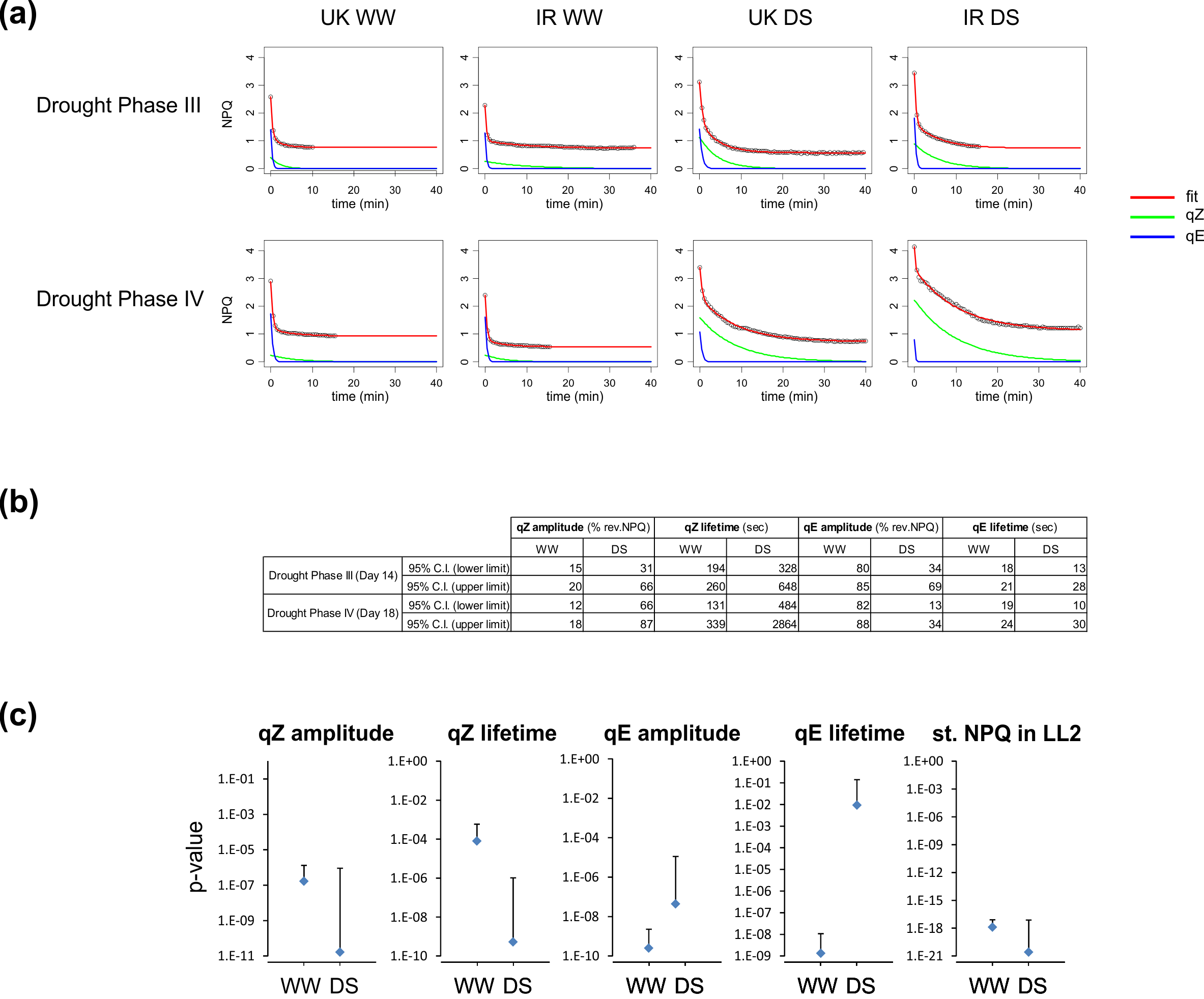

Supplement: Supplementary file 5 — Figure S4. Assessment of the quality of the NPQ decay fitting in FL analysis on UK and IR wheat cultivars (described in Figure S3c). [file PCE-43-1484-s005.tif]

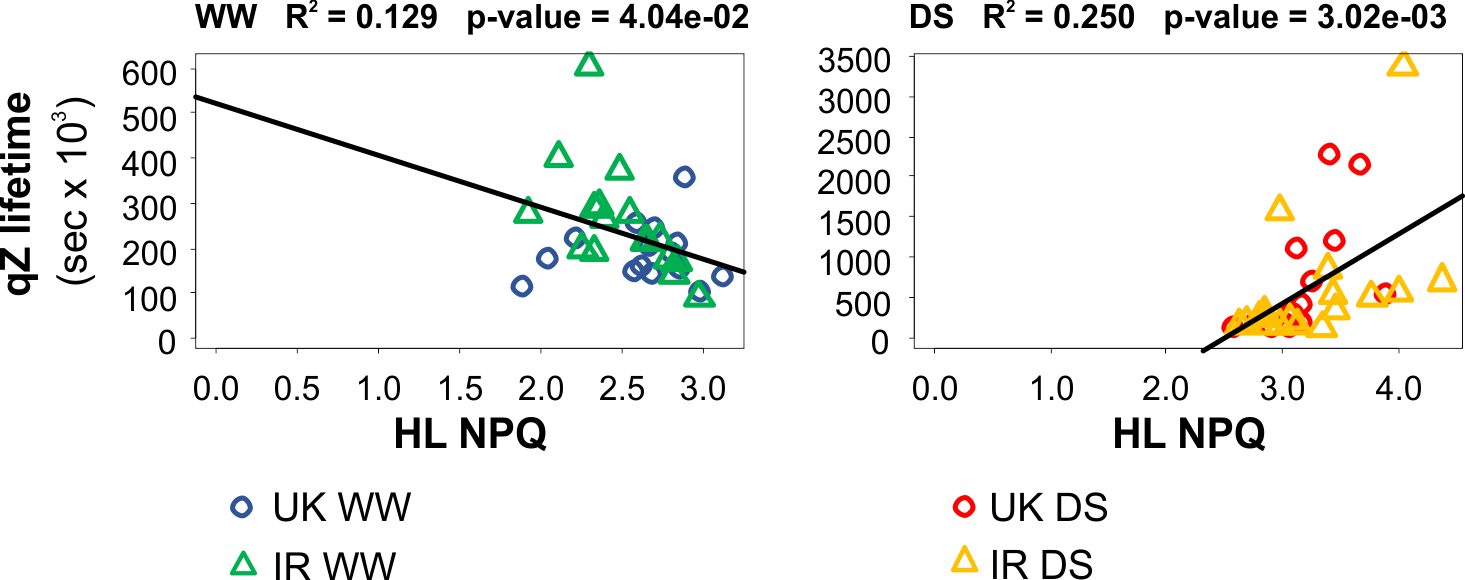

Supplement: Supplementary file 6 — Figure S5. Linear regression analysis of qZ lifetime versus high light‐induced NPQ (HL NPQ) in the UK and IR wheat cultivars in Drought Phases III and IV of the chamber experiment, based on the data shown in Figure 2. WW = well‐watered; DS = drought stressed. [file PCE-43-1484-s006.tif]

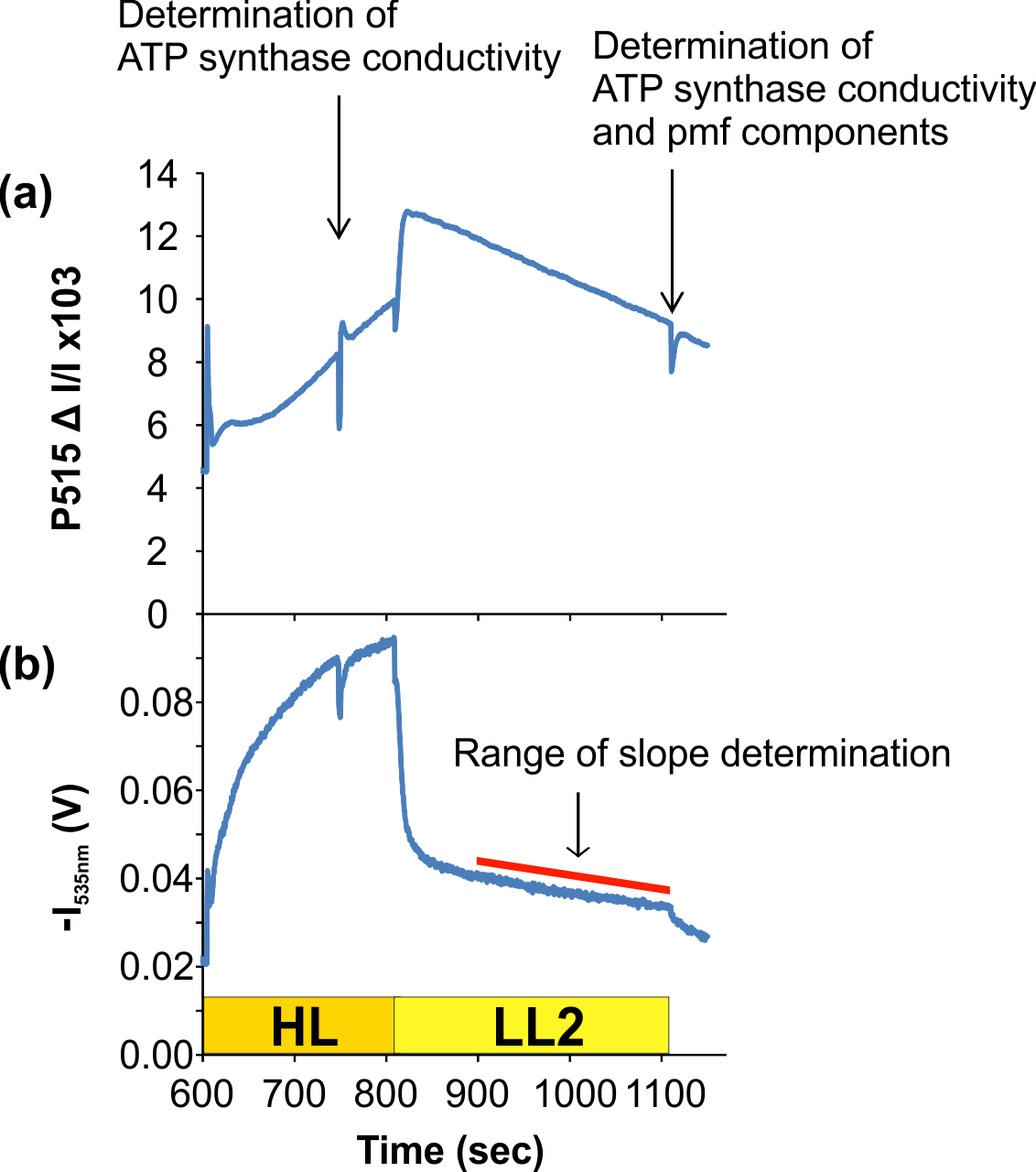

Supplement: Supplementary file 7 — Figure S6. Example of raw data for the determination of the parameters described in Figure 3d‐g. [file PCE-43-1484-s007.tif]

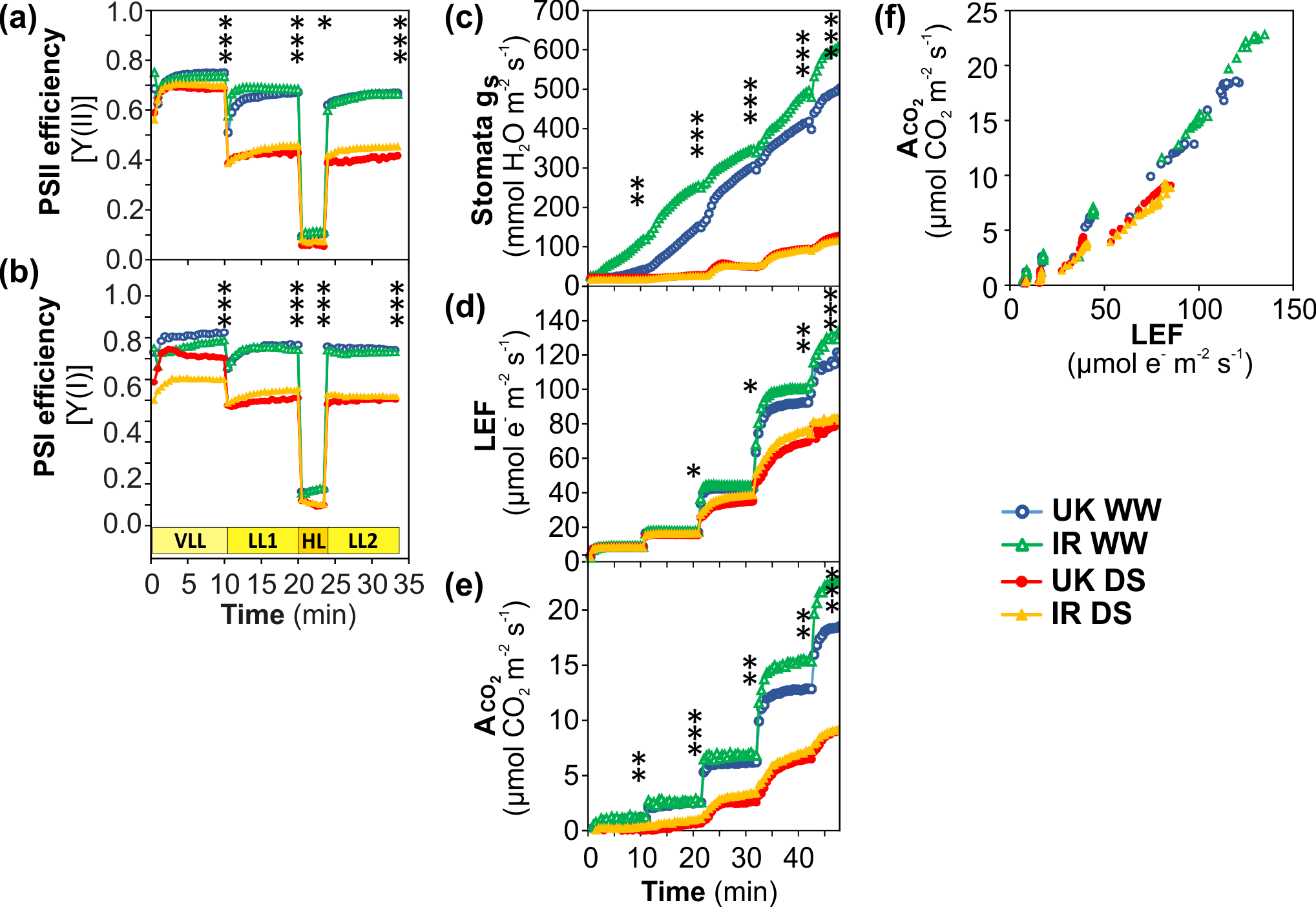

Supplement: Supplementary file 8 — Figure S7. Additional data on short‐term changes of photosynthetic activity in UK and IR wheat cultivars in the chamber experiment in Drought Phase III. [file PCE-43-1484-s008.tif]

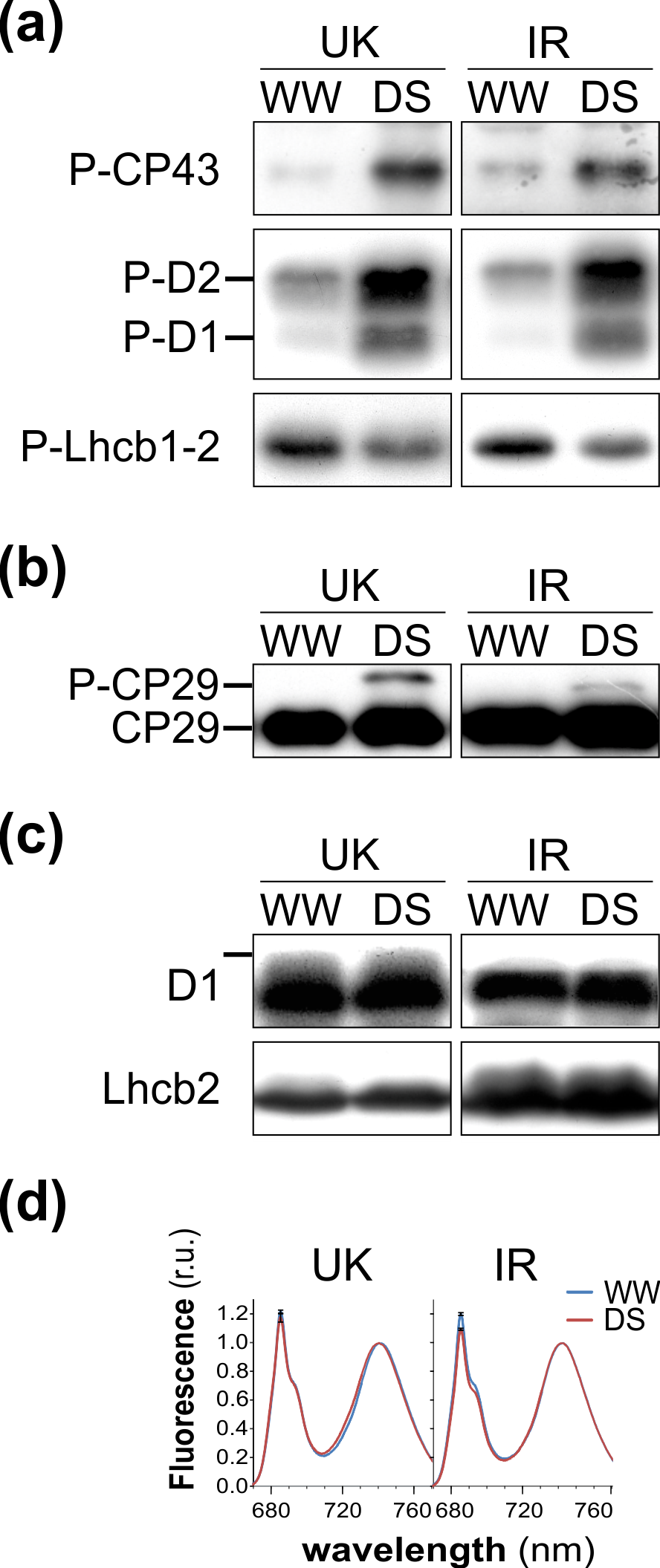

Supplement: Supplementary file 9 — Figure S8. Thylakoid protein phosphorylation and 77 K fluorescence emission spectra in UK and IR wheat cultivars in the Drought Phase IV of the chamber experiment. [file PCE-43-1484-s009.tif]
