# Supplementary material for: Adjustment of photosynthetic activity to drought and fluctuating light in wheat
Source: Plant Cell Environ. 2020 Mar 27;43(6):1484–500. doi: 10.1111/pce.13756 (PMC7384038; doi:10.1111/pce.13756)
Supplement: Supplementary file 10 — Table S1. List of model organisms obtained from KEGG (Kanehisa & Goto, 2000). [file PCE-43-1484-s010.docx]

**Table S1.** List of model organisms obtained from KEGG (Kanehisa and Goto, 2000). For these species, most characterized proteins belong to a KEGG Ontology (KO) group. HaMStR-OneSeq was run with the following parameters: -coreOrth=5, -minDist=genus, -maxDist=superkingdom, -strict, -checkCoorthologsRef, -global, -rep. Once an ortholog was identified, the tool annotates Pfam domains (Finn *et al.* 2010), SMART domains (Letunic, Doerks & Bork 2015) and low complexity regions in the protein sequence of both the seed and its ortholog. The resulting feature architectures are subsequently compared and a similarity score (FAS score) ranging from 0 to 1 is computed (FAS_seed-ortholog_) (Koestler, von Haeseler & Ebersberger 2010; Jain *et al.* 2018). Intuitively, the lower the FAS score, the fewer features two orthologs share, and the higher the likelihood that they have diverged in function. In the next step, we noted for each ortholog the KO group to which it has been assigned. We then computed the pair-wise FAS scores for each pair of members on the corresponding KO group, and computed the mean FAS score across all pairings (FASKO_mean). We then transferred the functional annotation of the KO group to the wheat seed protein only if FAS_seed-ortholog_ ≥ FASKO_mean. In cases where more than one KO annotation is represented by different orthologs of the seed protein, we considered all annotations in the downstream analyses. GO terms, where transferred to the KO, annotated wheat proteins by extracting the corresponding information from the KO / GO cross-reference mapping file provided by KEGG.

| *A. aeolicus* | *A. gossypii* | *A. nidulans* |
| --- | --- | --- |
| *A. pernix* | *A. thaliana* | *B. subtilis* |
| *C. albicans* | *C. elegans* | *C. hominis* |
| *D. melanogaster* | *D. rerio* | *E. coli* |
| *E. histolytica* | *H. pylori* | *H. sapiens* |
| *L. lactis* | *M. brevicollis* | *M. genitalium* |
| *M. jannaschii* | *M. musculus* | *M. tuberculosis* |
| *N. crassa* | *N. meningtidis* | *N. vectensis* |
| *P. falciparum 3D7* | *R. norvegicus* | *S. cerevisiae* |
| *S. pombe* | *Synechocystis sp.* | *T. brucei* |

**Table S2.** Phosphorylated proteins detected by mass spectrometry in the United Kingdom (UK) and Iran (IR) wheat cultivars. Variations of the protein content and the corresponding phospho-peptide in UK and IR plants under drought stress, relative to the respective control plants (in percentage), are indicated in the columns ‘UK DS’ and ‘IR DS’ (mean and SD), respectively. The mean of ‘UK DS’ and ‘IR DS’ values is reported in the ‘DS’ columns. p-values refer to the control vs drought comparison by two-way ANOVA analysis (0.01 < p < 0.05 is marked by *, 0.001 < p ≤ 0.01 by **, p < 0.001 by ***). In the ‘Phospho-site’ column the position of the phosphorylated residue in the protein sequence is reported. The modification of the phosphorylation level was estimated by taking into account the relative changes in the amount of phospho-peptide and the corresponding protein.

|  |  |  |  | |  | |  | | **Protein** | | | | | | | |  | | **Phospho-peptide** | | | | |  | |  | |  | | |  |  |
| --- | --- | --- | --- | --- | --- | --- | --- | --- | --- | --- | --- | --- | --- | --- | --- | --- | --- | --- | --- | --- | --- | --- | --- | --- | --- | --- | --- | --- | --- | --- | --- | --- |
| **Uniprot ID** | **Protein annotation** | **Phospho-site** | **DS** | **UK DS** | | | |  | | **IR DS** | |  | **DS** | **UK DS** | | | | | | |  | | | | | **IR DS** | |  | |  |  |  |
|  |  |  |  | **mean** | | **SD** | |  | | **mean** | **SD** |  |  |  | **mean** | | |  | | **SD** | | |  | | **mean** | | **SD** |  |  |  |  |  |
| P20858 | ATP synthase subunit beta (AtpB), chloroplastic | S496 | 3 | 10 | | 21 | |  | | -7 | 10 |  | -10 | -24 | |  | | | 26 | | | 5 | | | | | 29 | |  |  |  |  |
| A0A1D6B4Q8 | Calcium sensing receptor, chloroplastic | T368 | 24 | 20 | | 24 | |  | | 32 | 8 | * | -5 | -20 | |  | | | 27 | | | 9 | | | | | 31 | |  |  |  |  |
| A0A096UP27 | Cold induced 16 | S7 | 37 | 11 | | 42 | |  | | 62 | 30 | * | 27 | 106 | |  | | | 213 | | | 20 | | | | | 319 | |  |  |  |  |
|  |  | S8 |  |  | |  | |  | |  |  |  | 99 | 202 | |  | | | 235 | | | 86 | | | | | 453 | |  |  |  |  |
| A0A172WCB1 | Cold-responsive LEA/RAB-related COR protein | S17 | -17 | -17 | | 11 | |  | | -17 | 9 |  | -19 | -72 | |  | | | 25 | | | -4 | | | | | 1910 | |  |  |  |  |
| Q3S4I1 | Eukaryotic translation initiation factor 5A | S2 | 9 | -18 | | 18 | |  | | 13 | 33 |  | 7 | 346 | |  | | | 331 | | | -42 | | | | | 59 | |  |  |  |  |
|  |  | T4 |  |  | |  | |  | |  |  |  | -53 | -62 | |  | | | 67 | | | -45 | | | | | 219 | |  |  |  |  |
| W5BMK0 | Eukaryotic translation initiation factor 5A | S2 | 22 | 23 | | 97 | |  | | 22 | 54 |  | -1 | 3 | |  | | |  | | | -4 | | | | | 148 | |  |  |  |  |
|  |  | S4 |  |  | |  | |  | |  |  |  | 54 | 130 | |  | | | 227 | | | -1 | | | | | 22 | |  |  |  |  |
| A0A1D5S3M7 | Fructose-2,6-bisphosphatase | S278 | -22 | -29 | | 7 | |  | | -12 | 12 | *** | 20 | 33 | |  | | | 152 | | | 12 | | | | | 29 | |  |  |  |  |
| A0A0C4BK97 | Glycine cleavage system H protein, mitochondrial | S141 | 68 |  | |  | |  | | 68 | 244 |  | 14 | 40 | |  | | | 75 | | | 11 | | | | | 551 | |  |  |  |  |
| A0A1D5YQA8 | HMG-I/Y protein HMGa | S167 | 14 | 2 | | 30 | |  | | 23 | 14 |  | -73 | -84 | |  | | | 10 | | | 22 | | | | | 43 | |  |  |  |  |
| Q8LRU5 | HMG-Y-related protein A | S167 | 13 | -18 | | 30 | |  | | 30 | 28 |  | -9 | -8 | |  | | | 128 | | | -11 | | | | | 22 | |  |  |  |  |
| A0A1D6CET4 | Kinesin-like protein KIN-14L | S1062 | -5 | -19 | | 17 | |  | | 16 | 11 |  | 5 | -4 | |  | | |  | | | 8 | | | | | 112 | |  |  |  |  |
| A0A1D5XSR5 | Phosphoglucomutase, cytoplasmic | S180 | 9 | 3 | | 10 | |  | | 19 | 7 |  | 16 | 47 | |  | | | 82 | | | 6 | | | | | 87 | |  |  |  |  |
|  |  | T188 |  |  | |  | |  | |  |  |  | 49 | 16 | |  | | | 88 | | | 62 | | | | | 85 | |  |  |  |  |
| A0A1D6RID7 | Photosystem II subunit O (PsbO), chloroplastic | T110, S275 | 1 | 5 | | 12 | |  | | -1 | 13 |  | -49 | -56 | |  | | | 68 | | | -43 | | | | | 29 | |  |  |  |  |
| P69555 | Photosystem II subunit H (PsbH), chloroplastic | T3 | -7 |  | |  | |  | | -7 | 26 |  | 27 | 7 | |  | | | 88 | | | 45 | | | | | 68 | |  |  |  |  |
|  |  | T5 |  |  | |  | |  | |  |  |  | 166 | 109 | |  | | | 50 | | | 271 | | | | | 127 | |  |  |  |  |
| A0A1D5UI03 | Phototropin-2 | S493 | 0 | 13 | | 23 | |  | | -7 | 13 |  | -18 | -20 | |  | | | 30 | | | -17 | | | | | 98 | |  |  |  |  |
| A0A1D5ZWZ5 | Plastid movement impaired1;expressed protein | S100 | -7 | -16 | | 25 | |  | | 3 | 25 |  | 31 | 75 | |  | | | 206 | | | 11 | | | | | 29 | |  |  |  |  |
| A0A1D6D8M0 | Pyruvate, phosphate dikinase 1, chloroplastic | T464 | 20 | 12 | | 12 | |  | | 35 | 22 | * | 1446 | 893 | |  | | | 1705 | | | 3406 | | | | | 241 | |  |  |  |  |
| A0A1D5UR24 | Ribose 5-phosphate isomerase, type A protein | S6 | -45 | -45 | | 17 | |  | | -46 | 9 | *** | -17 | -38 | |  | | | 20 | | | 695 | | | | | 86 | |  |  |  |  |
| W5D591 | Small ubiquitin-related modifier | S2 | 19 | 27 | | 22 | |  | | 15 | 11 | ** | 2 | 43 | |  | | | 50 | | | -11 | | | | | 81 | |  |  |  |  |
| A0A1D6B773 | Tetratricopeptide repeat (TPR)-like superfamily protein | S1327 | -40 | -48 | | 29 | |  | | -28 | 30 | ** | 1370 | 452 | |  | | | 1123 | | | 2444 | | | | | 953 | |  |  |  |  |
|  |  | S1298 |  |  | |  | |  | |  |  |  | -51 | -61 | |  | | | 13 | | | -37 | | | | | 14 | | ** |  |  |  |

**Table S3.** Proteomics analysis on the United Kingdom (UK) and Iran (IR) wheat cultivars. ‘All proteins’ sheet includes a list of all the proteins identified. In ‘Dataset’ column, ‘full’ indicates the proteins that were identified in all samples, ‘not-full’ indicates proteins including missing values ‘Protein name’ column shows a common name for the protein. ‘Protein annotation’ reports the protein identification resulted from the bioinformatics analysis. In ‘DS’ column the relative changes (in percentage) of protein content in drought-stressed (DS) plants compared to control (WW) are reported, including both cultivars (In ‘DS’ column), and single cultivar analysis (‘UK DS’ and ‘IR DS’ columns). p-values refer to the control vs drought comparison by two-way ANOVA analysis (0.01 < p < 0.05 is marked in blue, 0.001 < p ≤ 0.01 in green, p < 0.001 in orange). ‘Selected proteins’ sheet includes a list of proteins selected from ‘All proteins’, including the proteins of **Figures 5 and 6**, ROS scavenging enzymes, and proteasome components.
