# Supplementary material for: Adjustment of photosynthetic activity to drought and fluctuating light in wheat
Source: Plant Cell Environ. 2020 Mar 27;43(6):1484–500. doi: 10.1111/pce.13756 (PMC7384038; doi:10.1111/pce.13756)
Supplement: Supplementary file 11 — Table S2. Phosphorylated proteins detected by mass spectrometry in the United Kingdom (UK) and Iran (IR) wheat cultivars. [file PCE-43-1484-s011.pdf]

Table S2. Phosphorylated proteins detected by mass spectrometry in the United Kingdom (UK) and Iran (IR) wheat cultivars. Variations of the protein content and the corresponding phospho-peptide in UK and IR plants under drought stress, relative to the respective control plants (in percentage), are indicated in the columns 'UK DS' and 'IR DS' (mean and SD), respectively. The mean of 'UK DS' and 'IR DS' values is reported in the 'DS' columns. p-values refer to the control vs drought comparison by two-way ANOVA analysis (0.01 < p < 0.05 is marked by *, 0.001 < p ≤ 0.01 by **, p < 0.001 by ***). In the 'Phospho-site' column the position of the phosphorylated residue in the protein sequence is reported. The modification of the phosphorylation level was estimated by taking into account the relative changes in the amount of phospho-peptide and the corresponding protein.

Uniprot ID	Protein annotation	Phospho-site	Protein						Phospho-peptide				
			DS	UK DS		IR DS			DS	UK DS		IR DS	
				mean	SD	mean	SD			mean	SD	mean	SD
P20858	ATP synthase subunit beta (AtpB), chloroplastic	S496	3	10	21	-7	10		-10	-24	26	5	29
A0A1D6B4Q8	Calcium sensing receptor, chloroplastic	T368	24	20	24	32	8	*	-5	-20	27	9	31
A0A096UP27	Cold induced 16	S7	37	11	42	62	30	*	27	106	213	20	319
		S8							99	202	235	86	453
A0A172WCB1	Cold-responsive LEA/RAB-related COR protein	S17	-17	-17	11	-17	9		-19	-72	25	-4	1910
Q3S4I1	Eukaryotic translation initiation factor 5A	S2	9	-18	18	13	33		7	346	331	-42	59
		T4							-53	-62	67	-45	219
W5BMK0	Eukaryotic translation initiation factor 5A	S2	22	23	97	22	54		-1	3		-4	148
		S4							54	130	227	-1	22
A0A1D5S3M7	Fructose-2,6-bisphosphatase	S278	-22	-29	7	-12	12	***	20	33	152	12	29

A0A0C4BK97	Glycine cleavage system H protein, mitochondrial	S141	68			68	244		14	40	75	11	551	
A0A1D5YQA8	HMG-I/Y protein HMGa	S167	14	2	30	23	14		-73	-84	10	22	43	
Q8LRU5	HMG-Y-related protein A	S167	13	-18	30	30	28		-9	-8	128	-11	22	
A0A1D6CET4	Kinesin-like protein KIN-14L	S1062	-5	-19	17	16	11		5	-4		8	112	
A0A1D5XSR5	Phosphoglucomutase, cytoplasmic	S180	9	3	10	19	7		16	47	82	6	87	
		T188							49	16	88	62	85	
A0A1D6RID7	Photosystem II subunit O (PsbO), chloroplastic	T110, S275	1	5	12	-1	13		-49	-56	68	-43	29	
P69555	Photosystem II subunit H (PsbH), chloroplastic	T3	-7			-7	26		27	7	88	45	68	
		T5							166	109	50	271	127	
A0A1D5UI03	Phototropin-2	S493	0	13	23	-7	13		-18	-20	30	-17	98	
A0A1D5ZWZ5	Plastid movement impaired1;expressed protein	S100	-7	-16	25	3	25		31	75	206	11	29	
A0A1D6D8M0	Pyruvate, phosphate dikinase 1, chloroplastic	T464	20	12	12	35	22	*	1446	893	1705	3406	241	
A0A1D5UR24	Ribose 5-phosphate isomerase, type A protein	S6	-45	-45	17	-46	9	***	-17	-38	20	695	86	
W5D591	Small ubiquitin-related modifier	S2	19	27	22	15	11	**	2	43	50	-11	81	
A0A1D6B773	Tetratricopeptide repeat (TPR)-like superfamily protein	S1327	-40	-48	29	-28	30	**	1370	452	1123	2444	953	
		S1298							-51	-61	13	-37	14	**
